# Ergosterol‐induced immune response in barley involves phosphorylation of phosphatidylinositol phosphate metabolic enzymes and activation of diterpene biosynthesis

**DOI:** 10.1111/nph.70022

**Published:** 2025-03-07

**Authors:** Pia Saake, Mathias Brands, Asmamaw Bidru Endeshaw, Sara Christina Stolze, Philipp Westhoff, Gerd Ulrich Balcke, Götz Hensel, Nicholas Holton, Cyril Zipfel, Alain Tissier, Hirofumi Nakagami, Alga Zuccaro

**Affiliations:** ^1^ Institute for Plant Sciences University of Cologne 50674 Cologne Germany; ^2^ Cluster of Excellence on Plant Sciences (CEPLAS) 50674 Cologne Germany; ^3^ Max Planck Institute for Plant Breeding Research Protein Mass Spectrometry 50829 Cologne Germany; ^4^ Heinrich Heine University Düsseldorf Institute for Plant Biochemistry 40225 Düsseldorf Germany; ^5^ Leibniz Institute for Plant Biochemistry 06120 Halle (Saale) Germany; ^6^ Heinrich Heine University Düsseldorf, Faculty of Mathematics and Natural Sciences Centre for Plant Genome Engineering 40225 Düsseldorf Germany; ^7^ The Sainsbury Laboratory University of East Anglia Norwich NR4 7UH UK; ^8^ Institute of Plant and Microbial Biology, Zurich‐Basel Plant Science Center University of Zurich 8008 Zurich Switzerland

**Keywords:** ergosterol, lipid signaling, pattern‐triggered immunity, phosphatidic acid, phosphatidylinositol phosphate, *Serendipita indica*

## Abstract

- Lipids play crucial roles in plant–microbe interactions, functioning as structural components, signaling molecules, and microbe‐associated molecular patterns (MAMPs). However, the mechanisms underlying lipid perception and signaling in plants remain largely unknown.
- Here, we investigate the immune responses activated in barley (*Hordeum vulgare*) by lipid extracts from the beneficial root endophytic fungus *Serendipita indica* and compare them to responses elicited by chitohexaose and the fungal sterol ergosterol. We demonstrate that *S. indica* lipid extract induces hallmarks of pattern‐triggered immunity (PTI) in barley. Ergosterol emerged as the primary immunogenic component and was detected in the apoplastic fluid of *S. indica*‐colonized barley roots. Notably, *S. indica* colonization suppresses the ergosterol‐induced burst of reactive oxygen species (ROS) in barley.
- By employing a multi‐omics approach, which integrates transcriptomics, phosphoproteomics, and metabolomics, we provide evidence for the phosphorylation of phosphatidylinositol phosphate (PIP) metabolic enzymes and activation of diterpene biosynthesis upon exposure to fungal lipids. Furthermore, we show that phosphatidic acid (PA) enhances lipid‐mediated apoplastic ROS production in barley.
- These findings indicate that plant lipids facilitate immune responses to fungal lipids in barley, providing new insights into lipid‐based signaling mechanisms in plant–microbe interactions.

## Introduction

Lipids are a diverse group of molecules that play crucial roles in plant nutrition, development, and plant–microbe interactions. As major constituents of the plasma and organelle membranes, they work in conjunction with the cell wall to establish the interface for environment interactions. Here, they act as structural elements, modulate physicochemical membrane properties, function as (stress) signaling molecules, and influence subcellular protein localization through lipid–protein interactions (Noack & Jaillais, 2020; Macabuhay *et al*., 2022; Zarreen *et al*., 2023).

Lipids are categorized into three main classes based on their chemical structures: sphingolipids, sterols, and (glycero)phospholipids (Moreau & Bayer, 2023). Among these, phosphoinositides stand out as crucial, low‐abundance signaling molecules, which are derived from phosphatidylinositol (PI), a ubiquitous phospholipid containing *myo*‐inositol in its head group. PI can be phosphorylated at various positions by phosphatidylinositol kinases (PIKs) to produce phosphatidylinositol phosphates (PIPs). In plants, phosphatidylinositol 4‐phosphate (PI4P) is the most abundant phosphoinositide, although PI3P, PI5P, and diphosphorylated forms, such as PI(4,5)P_2_ and PI(3,5)P_2_ have also been detected (Munnik & Vermeer, 2010).

During abiotic and biotic stress, PIPs can be interconverted and hydrolyzed to produce the signaling lipid phosphatidic acid (PA). PIPs are also associated with disease resistance (Xing *et al*., 2019; Qin *et al*., 2020), cytoskeletal rearrangements (Sinha *et al*., 2024), endo‐ and exocytosis (Synek *et al*., 2021; Marković & Jaillais, 2022), and the formation of membrane nanodomains (Gronnier *et al*., 2017; Jaillais & Ott, 2020). Nanodomains, accommodating membrane‐associated kinases, and receptor‐like kinases can act as signaling hubs during plant–microbe interactions to enable perception of microbe‐ or damage‐associated molecular patterns (MAMPs or DAMPs) and subsequent immune signaling (Couto & Zipfel, 2016; Jaillais & Ott, 2020).

Microbe‐associated molecular pattern recognition initiates a signaling cascade often involving an increase in cytosolic calcium concentration ([Ca^2+^]_cyt_), production of reactive oxygen species (ROS), and phosphorylation of mitogen‐activated protein (MAP) kinases (MAPKs). This cascade ultimately leads to altered gene expression and secretion of chemically diverse antimicrobial compounds, such as phytoalexins (Siebers *et al*., 2016; DeFalco & Zipfel, 2021). Altogether, this response is known as pattern‐triggered immunity (PTI). While plant lipids are important signaling molecules, microbial lipids can also be detected by plants as MAMPs. Lipid MAMPs can be directly recognized by plant receptors, as in the case of the medium‐chain‐3‐hydroxy fatty acids from *Pseudomonas syringae* lipopolysaccharides (Kutschera *et al*., 2019) or processed by secreted enzymes before recognition, such as ceramide D from *Phytophora infestans* (Kato *et al*., 2022).

The fungal sterol lipid ergosterol, a 5,7‐diene oxysterol, has also been shown to be perceived as a MAMP by plants (Kasparovsky *et al*., 2003). Sterols are core membrane components that regulate membrane organization, stability, and permeability (Macabuhay *et al*., 2022; Der *et al*., 2024). Ergosterol is the main sterol in most fungal membranes (Jordá & Puig, 2020) and is absent from plant membranes. It has been shown to induce early immune responses in various plant species. These responses include ROS accumulation in *Beta vulgaris* (Rossard *et al*., 2010), increase in [Ca^2+^]_cyt_ in *Nicotiana tabacum* (Kasparovsky *et al*., 2003; Vatsa *et al*., 2011), medium alkalinization in *B. vulgaris* and *N. tabacum* (Rossard *et al*., 2010; Vatsa *et al*., 2011), and induced expression of immunity‐related genes such as pathogenesis related (*PR*) genes and *WRKY* transcription factors in *Vitis vinifera*, *N. tabacum*, and *Solanum lycopersicum* (Laquitaine *et al*., 2006; Lochman & Mikes, 2006; Lindo *et al*., 2020). Despite being known as a MAMP for over two decades, the perception and signaling pathway of ergosterol remains largely unknown. This study investigates the molecular signaling mechanisms activated in barley (*Hordeum vulgare*) in response to lipid extracts, containing a mixture of lipids from the mycelium of the beneficial root endophytic fungus *Serendipita indica* (Sebacinales, Basidiomycota). Our data demonstrate that fungal lipids induce immunity in barley, with ergosterol identified as the primary immunogenic component of *S. indica* lipids and detected in the apoplast, the aqueous space between the host cells, of *S. indica*‐colonized barley roots. By integrating transcriptomics, phosphoproteomics, and metabolomics, we provide evidence that PIP signaling and diterpene biosynthesis are activated upon exposure to fungal lipids. Furthermore, we demonstrate that PA enhances lipid‐mediated apoplastic ROS production in barley. Notably, fungal colonization alters the host's phytosterol content and suppresses the ergosterol‐induced ROS burst, suggesting a counterstrategy against lipid‐mediated host immunity.

## Materials and Methods

### Plant material and growth conditions

Seeds of *Hordeum vulgare* L. cv Golden Promise were surface‐sterilized with 6% sodium‐hypochlorite for 1 h and washed six times for 30 min with sterile milliQ‐water. Seed coats were removed gently and seeds were germinated on a wet filter paper for 3 d in the dark at 21°C. Germinated seedlings were transferred to sterile WECK jars containing 100 ml solid 1/10 plant nutrition medium (PNM, pH 5.6) and 0.4% (w/v) Gelrite for MAMP treatments, 1.2% (w/v) Gelrite for fungal colonization, and grown under long‐day conditions (16 h : 8 h, 22°C : 18°C, day : night cycle, light intensity of 108 μmol m^−2^ s^−1^) for an additional 4 d for MAMP treatment, and 3‐, 7‐ and 14‐d for fungal colonization assays. For Ca^2+^‐influx assays, barley lines expressing apoaequorin (Hv^AEQ^) were generated (Supporting Information Methods S1).

*Solanum lycopersicum* L. cv Moneymaker wild‐type (WT) and *serk3a serk3b* mutant seeds (Methods S1) were sown on soil and grown for *c*. 3 wk in the glasshouse under long‐day conditions (17 h : 7 h, light : dark, 25–28°C, *c*. 35–40% humidity). For ROS assays, 3‐mm leaf disks of the two youngest adult leaves of 3‐wk‐old tomato plants were used.

Seeds of a *Nicotiana benthamiana* D. line expressing aequorin (Nb^AEQ^) (Wanke *et al*., 2020) were sown on soil and grown for *c*. 3 wk in the glasshouse under long‐day conditions (16 h : 8 h, 22°C : 25°C, day : night cycle, light intensity of *c*. 140 μmol m^−2^ s^−1^, maximal humidity of 60%). For ROS and Ca^2+^‐influx assays, 3‐mm leaf disks of the youngest adult leaf of 3‐wk‐old plants were used.

Seeds of *Arabidopsis thaliana* L. Col‐0 plants expressing cytosolic apoaequorin (At^AEQ^) were surface sterilized with EtOH and subsequently dried under the clean bench. Dried seeds were placed on ½ Murashige Skoog (MS medium, with vitamins, pH 5.7) plates containing 0.5% sucrose and 0.4% (w/v) Gelrite and stratified for 2–5 d at 4°C in the dark. Subsequently, plants were grown in short‐day conditions (8 h : 16 h, 22°C : 18°C, light : dark, with 130 μmol m^−2^ s^−1^ of light) for 7–8 d. For ROS assays, one seedling was transferred into each well of a 96‐well plate. For Ca^2+^‐assays, seedlings were transferred to 24‐well plates containing 1 ml ½ MS medium + sucrose and grown for another 4 d before use in the assay.

### Cultivation of *S. indica* and plant colonization

Cultivation and chlamydospore isolation of *S. indica* (DSM11827) was done as previously described (Wawra *et al*., 2016; Sarkar *et al*., 2019).

Germinated barley seedlings were prepared as described previously and inoculated with 3 ml of either sterile water as control or *S. indica* chlamydospores (500 000 spores ml^−1^). Roots were harvested at 3‐, 7‐, and 14 d post inoculation (dpi), washed thoroughly in ice‐cold water to remove extraradical fungal hyphae, briefly dried on tissue paper and frozen in liquid nitrogen. Four barley plants were used per jar and pooled per biological replicate.

### ROS accumulation assay

Reactive oxygen species assays were performed as described previously (Chandrasekar *et al*., 2022). Preparation of *N. benthamiana*, tomato, and *A. thaliana* material is described previously. For barley, roots and shoots of 7‐d‐old seedlings were separated. Three root pieces (0.5 cm) or one 3‐mm leaf disk were transferred to each well of a 96‐well microtiter plate (white, flat bottom) containing 200 μl of 2.5 mM 2‐(N‐morpholino)ethanesulfonic acid (MES) buffer, pH 5.6. The plate was incubated overnight (ON) at 21°C for recovery. The next day, the buffer was replaced with 100 μl 2.5 mM MES buffer containing 20 μM LO‐12 and 20 μg ml^−1^ Horseradish peroxidase (HRP). After 25‐min incubation in the dark, 100 μl twofold concentrated elicitor solution or solvent control was added to each well, and chemiluminescence was measured using a TECAN SPARK 10 M microplate reader over all wells for 2 h with an integration time of 450 ms.

### Calcium influx assay

Preparation of *N. benthamiana* and *A. thaliana* material is described previously. For barley, roots and shoots were prepared as described for ROS accumulation assays but Hv^AEQ^ plants were used. Before ON recovery, the buffer in the wells was replaced with 100 μl 2.5 mM MES buffer containing 10 μM coelenterazine and 10 mM CaCl_2_ per well and plates were incubated ON in the dark at 21°C. The next day, chemiluminescence was measured using a TECAN SPARK 10 M microplate plate reader. After the baseline measurement (5 min), 100 μl of twofold concentrated elicitor solution was added manually. Photon emission was constantly measured for 30 min. Subsequently, 100 μl of discharge solution (3 M CaCl_2_ in 50% EtOH) was injected into each well, followed by constant measurement for 1 min. All steps were performed with an integration time of 450 ms. In all assays, two columns (16 wells) were measured per run.

### MAPK phosphorylation

Barley root segments and leaf disks were prepared as described previously for ROS and Ca^2+^‐influx assays. Twenty‐four randomly selected pieces were transferred into each well of a 24‐well plate containing 1 ml 2.5 mM MES buffer. Plates were incubated ON at 21°C for recovery. The next day, 500 μl buffer of each well were removed and replaced with twofold concentrated elicitor solution and gently mixed. At 5‐, 10‐, 20‐, or 30‐min post treatment, roots were removed from the treatment solution, gently dried on a tissue paper, snap‐frozen in liquid nitrogen, and homogenized with glass beads in the TissueLyserII (Qiagen) for four times 30 s, 30 Hz in ice‐cold holders. Phosphorylated proteins were isolated using phosphoprotein extraction buffer (50 mM Tris–HCl (pH 7.5), 2 mM DTT, 5 mM EDTA, 5 mM ethylene glycol‐bis(β‐aminoethyl ether)‐N,N,N′,N′‐tetraacetic acid (EGTA), 10 mM NaF, 50 mM β‐glycerolphosphate, 10% glycerol, 1 tablet each of Roche Complete Mini – EDTA‐free and PhosStop phosphatase inhibitor per 10 ml). Protein concentration was determined using the Bradford Assay following the manufacturer's instructions, and 4 μg protein of each sample was separated on sodium dodecyl sulfate‐polyacrylamide gel electrophoresis (SDS‐PAGEs) and subsequently transferred to nitrocellulose membranes. Membranes were blocked for 1 h with 2.5% Bovine serum albumin in Tris‐buffered saline with Tween20 (BSA TBS‐T) and incubated ON with the primary antibody (anti‐p44/p42, 1 : 1500) in 2.5% TBS‐T BSA at 4°C. The next day, the membranes were washed with 1× TBS‐T, and the secondary antibody (anti‐rabbit IgG, 1 : 50 000) was added for 1 h. After washing with 1× TBS‐T and 1× TBS, blots were developed using 1 ml SuperSignal™West Femto (Thermo Fisher Scientific, Waltham, MA, USA) solution per membrane. Western blots were imaged using the Fujifilm LAS 4000 mini camera.

### Elicitor preparations

The following chemicals were used as elicitors, pre‐ or cotreatments: chitohexaose (O‐CHI6; Megazyme, Bray, Ireland), flg22 peptide (RP19986; GenScript, Piscataway, NJ, USA), ergosterol pharmaceutical standard (PHR1512; Supelco (Merck, Darmstadt, Germany), L‐α‐phosphatidylinositol (soy PI, Avanti, 840 044), L‐α‐phosphatidic acid (egg PA, Avanti, 840 101), L‐α‐phosphatidylcholine (soy PC, Avanti, 441 601), L‐α‐phosphatidylinositol 4‐monophosphate (P9638; Sigma‐Aldrich), L‐α‐phosphatidylinositol 4,5‐diphosphate (P9763; Sigma‐Aldrich). Stock solutions of lipids were prepared in methanol and used to prepare elicitor solutions of appropriate concentrations in aqueous MES buffer as described later. Self‐produced lipid extracts and lipid fractions were evaporated with N_2_ gas and resuspended in MeOH as solvent. As control, the respective solvent was processed in the same way. All elicitor and control solutions were prepared as twofold concentrated solutions in 2.5 mM MES buffer, pH 5.6 containing 1 : 20 (v/v %) MeOH. For liposomes, PA was evaporated under N_2_ gas and the lipid film hydrated with buffer (25 mM HEPES, pH = 7.5, 50 mM KCL, 1 mM MgCL_2_) at 4°C, rapidly vortexed for 30 s and sonicated on ice five times for 10 s with each 10 s pause before use in cotreatment ROS assay with ergosterol.

### RNA sequencing and quantitative reverse transcription polymerase chain reaction

For RNA sequencing (RNA‐seq) and quantitative reverse transcription polymerase chain reaction, barley roots were prepared as described for the MAPK phosphorylation assay. RNA extraction, cDNA synthesis, and quantitative reverse transcription polymerase chain reaction were performed as described previously (Sarkar *et al*., 2019).

For RNA‐seq, 25 μl RNA with a concentration of 100 ng μl^−1^ was used. For Illumina‐compatible RNA‐Seq libraries, at first an enrichment for poly‐A RNAs was performed (NEBNext® Poly(A) mRNA Magnetic Isolation Module; New England Biolabs, Ipswich, MA, USA), followed by library generation with NEBNext Ultra™II Directional RNA Library Prep Kit for Illumina (New England Biolabs). Next, sequencing‐by‐synthesis was done on a NextSeq 2000 device in 2 × 150 bp paired‐end read mode Library construction, and sequencing was performed at the Genome Centre of the Max Planck Institute for Plant Breeding, Cologne. For information on processing of the sequencing data, see Methods S1.

### Apoplastic fluid isolation of barley roots

Barley seedlings were grown and inoculated with *S. indica* on 1/10 PNM as described previously. Per replicate, 110 barley seedlings were used, which yielded *c*. 1 ml apoplastic fluid per replicate. For extraction of apoplastic fluid, the roots were washed thoroughly in ice‐cold water to remove external fungal hyphae and cut into 2‐cm pieces. Root pieces were submitted to five cycles of vacuum infiltration (15 min 250 mbar, 1.5 min ATM) in ice‐cold water. Subsequently, roots were dried on tissue paper and transferred into a 20‐ml syringe inside a 50‐ml falcon tube and centrifuged for 15 min at 4°C, 711 ***g***, lowest de‐ and acceleration to collect apoplastic fluid (*c*. 1–2 ml per replicate) in the bottom of the falcon tube. Apoplastic fluid was stored on ice at 4°C until further use. Roots were flash‐frozen in liquid nitrogen and stored at −80°C until further use.

### Lipid extraction and fractionation by solid‐phase extraction

Lipid extraction was done according to the method described (Bligh & Dyer, 1959) using chloroform : methanol : formic acid (1 : 1 : 0.2 v/v). To extract lipids from *S. indica* mycelium or colonized or mock‐inoculated barley roots, frozen material was ground in liquid nitrogen into a very fine powder using a mortar and pestle. Per sample, *c*. 200 mg of homogenized plant or fungal tissue was used. For fractionation of crude lipids, the lipid extract was separated by two consecutive solid‐phase extractions (SPE) using Strata®SI‐1 (55 μm, 70 Å, 1 ml) silica columns. In the first SPE, lipids were separated via elution with chloroform (CHCL_3_) and MeOH into neutral (CHCL_3_) and polar lipids (MeOH). The CHCL_3_ fraction was evaporated under N_2_ gas and used for further fractionation of the neutral lipids using a hexane:diethylether gradient on a second silica column as described previously (vom Dorp *et al*., 2013) (Methods S1). All fractions that were used in immunity assays were dried under N_2_ and resuspended in 4 ml MeOH before use.

### Phosphoproteomics

To analyze phosphorylated proteins in response to elicitor treatments, barley roots were treated as described for the MAPK phosphorylation assay, and root material was harvested 10 min post treatment. Root material was ground into a very fine powder, and phospho‐enriched proteins were extracted using 1 ml extraction buffer (8 M urea, 20 μl ml^−1^ Phosphatase Inhibitor Cocktail 2 (P5726‐5ML; Sigma), 20 μl ml^−1^ Phosphatase Inhibitor Cocktail 3 (P0044‐5ML; Sigma), 5 mM DTT), alkylated with CAA (550 mM stock, 14 mM final) and the reaction was quenched with DTT (5 mM final). An equivalent of 500 μg total protein per sample was diluted to 1 M urea with 100 mM Tris–HCl pH 8.5, 1 mM CaCl_2_ and samples were digested with 5 μg LysC (stock: 1 μg μl^−1^ Lys‐C (WAKO) in 50 mM NH_4_HCO_3_) for 4 h at RT and 5 μg trypsin (stock: 1 μg μl^−1^ in 1 mM HCl) ON at 37°C. After incubation, samples were acidified with TFA to 0.5% final concentration, and samples were desalted using C18 SepPaks (1 cc cartridge, 100 mg (WAT023590)). Phosphopeptide enrichment was performed by hydroxy acid‐modified metal‐oxide chromatography (HAMMOC) (adapted from: Nakagami, 2014). Finally, the samples were dried in a vacuum evaporator, and dissolved in 10 μl 2% ACN, 0.1% TFA (A* buffer) for MS analysis.

Samples were analyzed using an Ultimate 3000 RSLC nano (Thermo Fisher) coupled to an Orbitrap Exploris 480 mass spectrometer equipped with a FAIMS Pro interface for Field asymmetric ion mobility separation (Thermo Fisher). Raw data were processed using the MaxQuant software (v.1.6.3.4) (Cox & Mann, 2008) with label‐free quantification (LFQ) and iBAQ enabled (Tyanova *et al*., 2016). For more details on sample‐ and data processing, see Methods S1.

### Measurements of sterols via GC‐TOF‐MS

For sterol measurement via GC‐TOF‐MS, 5 nmol stigmastanol was added as internal standard in a CHCL_3_:MeOH (2 : 1) mix before lipid extraction. Lipid extraction and SPE were performed as described previously. Dried free sterol fractions (50 μl) were directly derivatized with 100 μl MSTFA for 30 min at 80°C. Subsequently, the samples were transferred to glass vials and measured with split ratio of 1 : 10. To measure crude lipid extracts of apoplastic fluid, dried lipid extracts were resuspended in 300 μl hexane and split in 2× 150 μl samples. One 150 μl sample each was evaporated under N_2_ gas. Dried samples were derivatized with MSTFA for 30 min at 80°C automatically before measurement using a Multipurpose Autosampler (Gerstel). One microlitre of sample was injected with an automatic liner exchange system in conjunction with a cold injection system in splitless mode (ramping from 50°C to 250°C at 12°C s^−1^) into the GC with a helium flow of 1 ml min^−1^. Chromatography was performed using a 7890B GC system (Agilent Technologies, Santa Clara, CA, USA) with a HP‐5MS column with 5% phenyl methyl siloxane film (Agilent 19091S‐433, 30 m length, 0.25 mm internal diameter, 0.25 μM film). Compounds were identified via MassHunter Qualitative (v b08.00; Agilent Technologies) by comparison of spectra to the NIST14 Mass Spectral Library. Ergosterol was verified using a pharmaceutical standard (PHR1512; Supelco) and stigmastanol was used as an internal standard. Peaks were integrated using MassHunter Quantitative (v b08.00; Agilent Technologies). For relative quantification, all metabolite peak areas were normalized to the corresponding peak area of the internal standard stigmastanol and the sample fresh weight (mycelium, roots) or volume (AF).

### Diterpene measurements via LC‐MS

Germinated barley seedlings were treated with solvent control, 250 nM ergosterol or inoculated with *Bipolaris sorokiniana* spores (5000 spores ml^−1^, 5 ml) and grown for 6 d as described above. To harvest root exudates, plants were removed from the medium and washed gently in 25 ml water to wash off residual diterpenes attached on the outside the roots. The medium was collected with the wash water from the roots and flash‐frozen in liquid nitrogen. Diterpenes were extracted as described previously (Liu *et al*., 2024). For information on LC‐MS measurement, see Methods S1.

## Results

### Lipids from the beneficial endophyte *S. indica* induce immunity in barley

**Fig. 1:**
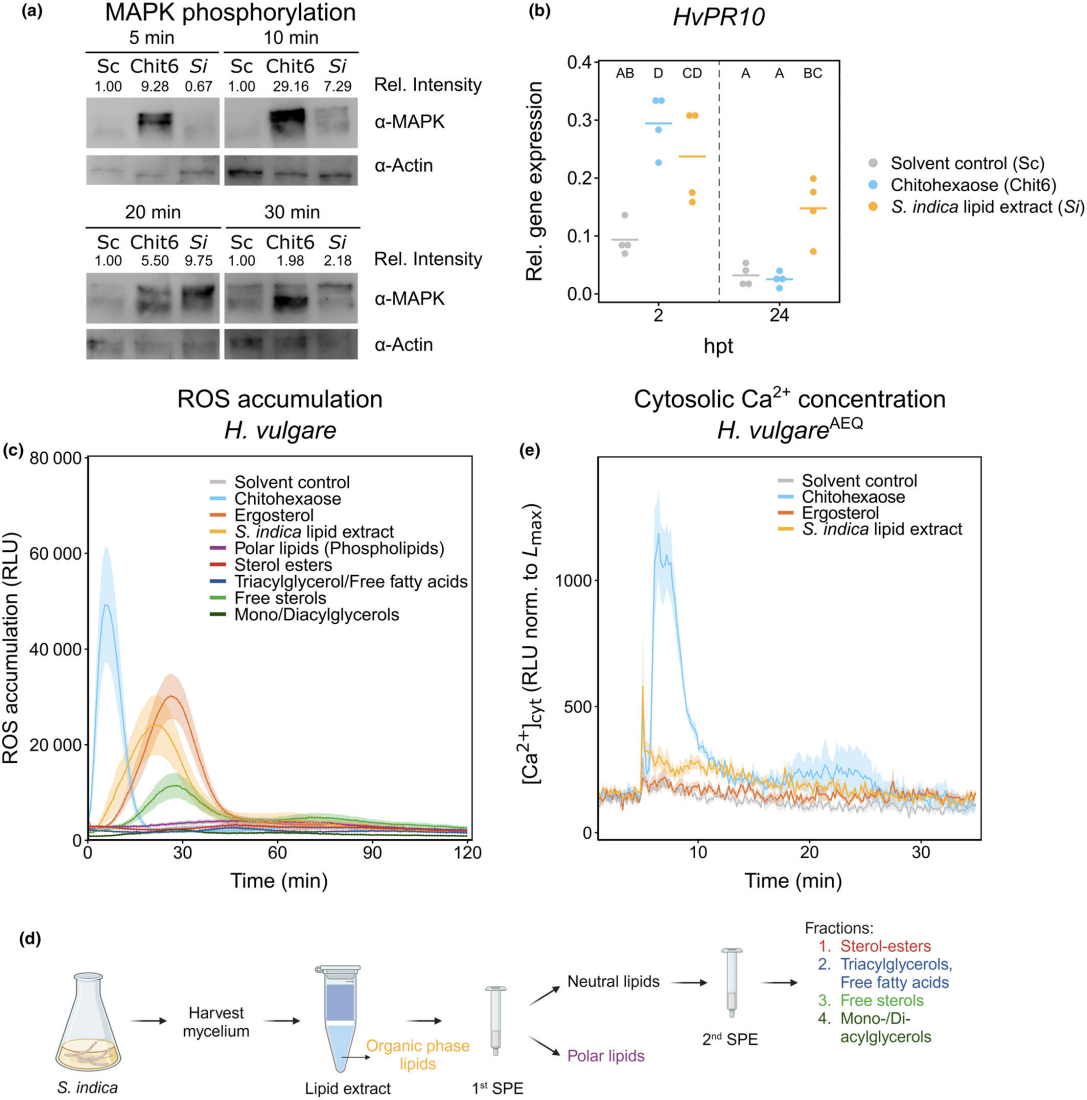
Lipids from the beneficial root endophyte *Serendipita indica* induce immunity in barley roots. (a) Mitogen activated protein kinase (MAPK) phosphorylation in roots of 7‐d‐old barley plants, treated with the indicated microbe‐associated molecular patterns (MAMPs) or solvent control for 5‐, 10‐, 20‐ or 30‐min. 1^st^ antibody, phospho‐p44/42 MAPK (upper) or α‐Actin as loading control (lower); 2^nd^ antibody, anti‐rabbit IgG. Sc, solvent control [1 : 160]; Chit6, chitohexaose [250 nM]; *Si*, *S. indica* lipid extract [1 : 160]. Relative Intensity depicts the MAPK signal intensity normalized to the Actin signal intensity of the same treatment and to the solvent control (Sc) for each time point. The experiment was repeated four times with similar results (Supporting Information Fig. S2). (b) Gene expression of *HvPR10* relative to the housekeeping gene *HvUBI* in barley roots determined by quantitative reverse transcription polymerase chain reaction. Roots were treated with the indicated MAMPs or solvent control for 2 or 24 h. Horizontal lines depict mean values. Letters indicate significant differences based on ANOVA + *post hoc* Tukey test (*P* ≤ 0.05). (c, e) Reactive oxygen species (ROS) accumulation (c) and cytosolic Ca^2+^ concentration ([Ca^2+^]_cyt_) (e) in roots of 7‐d‐old barley plants, treated with the indicated lipid fractions or solvent control as negative control. Values represent means (normalized to maximum luminescence (*L*_max_) over all wells for Ca^2+^) ± SEM from six to eight wells, each containing three root pieces. The following concentrations and dilutions were used: *Serendipita indica* total lipid extract and fractions, 1 : 160 (v/v); ergosterol, 250 nM; chitohexaose, 25 μM. All treatments contained a final amount of 1 : 40 (v/v) methanol. (d) Lipid fractionation method. This figure was created using BioRender: https://BioRender.com/v58k798. hpt, hours post treatment; RLU, relative luminescence unit; ROS, reactive oxygen species; SPE, solid‐phase extraction.

To investigate the immune response of barley to fungal lipids, root and leaf tissues were treated with a crude lipid extract from *S. indica* mycelium grown in axenic culture. MAPK activation, *PR* gene induction, and Ca^2+^‐ and ROS burst production were monitored. The fungal MAMP chitohexaose (Chit6) served as a positive control, while a solvent control (Sc) was used as the negative control. Treatment with *S. indica* lipid extract (*Si*) induced MAPK phosphorylation in barley roots 10–20 min post treatment with the highest signal after 20 min (Fig. 1a). This induction was weaker and occurred later than with chitohexaose treatment, which resulted in MAPK phosphorylation as early as 5 min post treatment (Fig. 1a). The expression of the immunity‐related gene *HvPR10* (Mahdi *et al*., 2022) was induced to a similar extent upon treatment with *S. indica* lipid extract and chitohexaose 2 h post treatment (hpt) (Fig. 1b). While *HvPR10* expression in chitohexaose‐treated roots returned to baseline levels after 24 h, expression in *S. indica* lipid extract‐treated roots remained high at this time point (Fig. 1b). In barley leaves, both chitohexaose and *S. indica* lipid extract treatments induced *HvPR10* expression at 24 hpt (Fig. S1A). In response to MAMP treatment, the level of apoplastic ROS increases rapidly, followed by a rapid decline. Treatment with the lipid extract of *S. indica* induced a ROS burst in barley roots and leaves, which reached a maximum *c*. 20 min post elicitor treatment. By contrast, the chitohexaose‐induced ROS burst showed a more rapid increase, reaching a maximum at *c*. 8 min, followed by a steep decline (Figs 1c, S1B).

To identify the immunogenic components of the *S. indica* lipid extract, we fractionated the crude lipid extract by SPE (Fig. 1d). We found that the ROS burst correlated with the fraction containing free sterols, as all other fractions did not induce ROS bursts in barley roots (Fig. 1c). The timing and shape of the ROS burst induced by the sterol fraction closely matched those induced by the total lipid extract of *S. indica* and a pharmaceutical ergosterol standard (Fig. 1c, yellow and orange) and differed from the ROS burst induced by chitohexaose (Fig. 1c, light blue). Consistent with this, ergosterol treatment also induced MAPK phosphorylation in barley roots with the highest signal obtained at 20 min post treatment (Fig. S2).

Another hallmark of early immune signaling is an increase in cytosolic Ca^2+^ concentration ([Ca^2+^]_cyt_). While chitohexaose induced a strong and rapid Ca^2+^‐burst, only a slight increase in [Ca^2+^]_cyt_ was observed when barley roots were treated with ergosterol or *S. indica* lipids (Fig. 1e).

To investigate whether the perception of *S. indica* lipids occurs in other plant species, ROS‐ and Ca^2+^‐burst assays were performed in *N. benthamiana* and *A. thaliana*. In *N. benthamiana* leaves, *S. indica* lipids induced a pronounced ROS burst accompanied by a minor yet distinct increase in [Ca^2+^]_cyt_ (Fig. S3A,B). Timing and shape of the *S. indica* lipid‐induced ROS burst overlapped with the ergosterol‐induced ROS burst (Fig. S3C). In *A. thaliana* seedlings, total ROS accumulation increased after treatment with *S. indica* lipids or ergosterol; however, no pronounced peak was observed (Fig. S3D), and only a slight increase in [Ca^2+^]_cyt_ was detected (Fig. S3E).

Taken together, these results indicate that *S. indica* lipid extract induces MAPK phosphorylation, *PR* gene expression, and ROS burst in barley, which are typical hallmarks of PTI. In addition, the perception of fungal lipids is conserved between barley and *N. benthamiana*, in leaves. In contrast to chitohexaose, *S. indica* lipid‐induced ROS accumulation is not accompanied by a strong Ca^2+^‐burst but rather appears to be independent of it.

### Ergosterol is the primary immunogenic component of *S. indica* lipid extract and is present in the apoplast of colonized barley roots

**Fig. 2:**
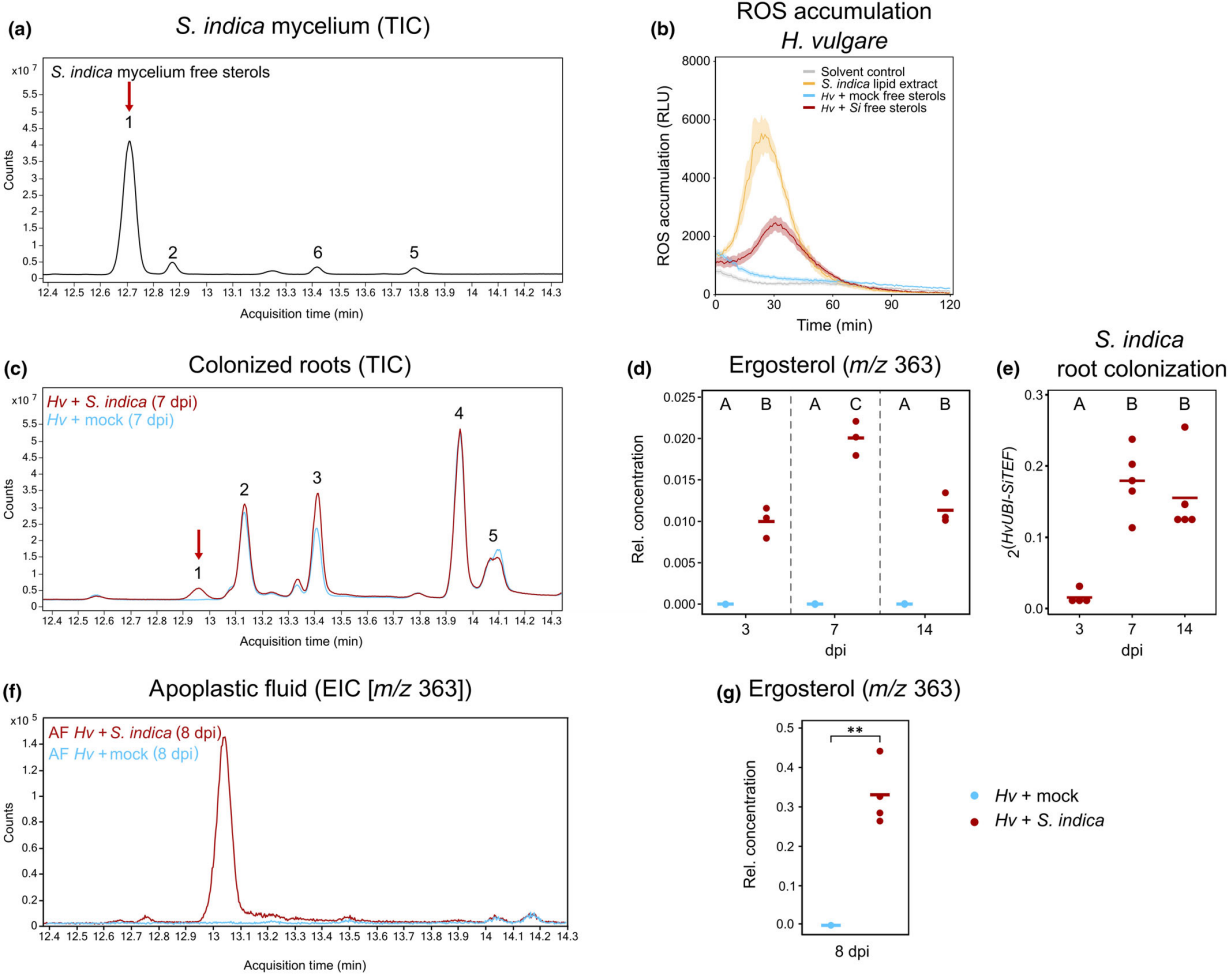
Ergosterol is present in the apoplast of colonized barley roots and is the main immunogenic component of *Serendipita indica* lipid extract. (a, c) gas chromatography/time‐of‐flight mass spectrometry (GC‐TOF‐MS) total ion chromatograms (TICs) of the free sterol fraction from *S. indica* lipids isolated from mycelium (a) or whole root samples of mock‐treated or *S. indica*‐colonized barley roots (c). Major peaks in (a) and (c) are labeled in the following order: 1, Ergosterol; 2, Ergosta‐7,22‐dien‐3ol / Campesterol; 3, Stigmasterol; 4, β‐Sitosterol; 5, Stigmastanol (internal standard); 6, Ergost‐8(14)‐en‐3‐ol. (b) Reactive oxygen species (ROS) accumulation in roots of 7‐d‐old barley plants, treated with *S. indica* lipid extract or sterol fractions isolated from mock‐treated or *S. indica*‐colonized barley roots at 7 d post inoculation (dpi) or solvent control as negative control. Values represent means ± SE from eight wells, each containing three root pieces. *S. indica* total lipid extract and fractions were used in 1 : 160 (v/v) dilution. All treatments contained a final amount of 1 : 40 (v/v) methanol. (d) Quantification of ergosterol in sterol fractions of *S. indica*‐colonized barley roots at 3‐, 7‐ and 14 dpi. (e) *Serendipita indica* intraradical colonization of barley roots determined as relative expression of the fungal housekeeping gene *SiTEF* to the barley housekeeping gene *HvUBI* at 3‐, 7‐, and 14 dpi. Data were obtained from an independent experiment performed the same way as for (d). (f) Extracted ion chromatogram (EIC) of ergosterol (*m/z* 363) in total lipid extract of apoplastic fluid of mock‐treated or *S. indica*‐colonized barley roots (8 dpi). Representative image of one replicate. Quantification of four replicates is shown in (g). Horizontal lines in (d, e, g) depict mean values. Letters indicate significant differences based on ANOVA + *post hoc* Tukey test (*P* ≤ 0.05). Asterisks indicate significant difference based on Student's *t*‐test: **, *P* ≤ 0.01 . Relative ergosterol concentration was calculated based on internal standard response and normalized to sample fresh weight (d) or ml apoplastic fluid (g). *Hv*, *Hordeum vulgare* (barley); RLU, relative luminescence unit; ROS, reactive oxygen species; *Si*, *Serendipita indica*.

Since the free sterol fraction elicited a comparable ROS burst as the crude *S. indica* lipid extract in barley (Fig. 1c), we analyzed this fraction by GC‐TOF‐MS to identify the major sterols potentially triggering the immune response. We found that ergosterol was the major lipid in this fraction. In addition, smaller amounts of related sterols, presumably ergosta‐7,22‐dien‐3‐ol and ergost‐8(14)‐en‐3‐ol were detected (Fig. 2a).

Microbes often employ different strategies to avoid MAMP recognition by the host. For example, reducing abundance or shielding of the MAMP during colonization (van Boerdonk *et al*., 2024). To investigate whether ergosterol is present during endophytic root colonization, total lipids were isolated from mock‐treated or *S. indica*‐colonized barley roots at 3‐, 7‐ and 14 days post inoculation (dpi). Subsequently, free sterols were isolated using SPE, following the protocol previously described for extracting lipids from *S. indica* mycelium (Fig. 1d). Consequently, samples from mock‐treated roots contained free sterols derived exclusively from plant roots, whereas samples from colonized roots comprised a mixture of plant and fungal free sterols. Roots were thoroughly washed before extraction to remove extraradical mycelium. The free sterol fractions were then tested for their potential to elicit ROS production in barley roots (Fig. 2b). Free sterols isolated from colonized barley roots, but not from mock‐treated barley roots, induced a ROS burst with similar timing to that obtained with *S. indica* mycelial lipids (Fig. 2b). GC‐TOF‐MS analysis confirmed the presence of ergosterol in colonized barley roots (Fig. 2c). Since mock‐inoculated roots lack ergosterol (Fig. 2c), and no other major qualitative differences in identified free sterols were observed using our method, the ROS burst associated with free sterols isolated from *S. indica*‐colonized roots is most likely attributable to ergosterol. The relative amount of ergosterol in *S. indica*‐colonized roots increased from 3 to 7 dpi, followed by a decrease at 14 dpi (Fig. 2d). Intraradical *S. indica* colonization, determined by expression of the fungal housekeeping gene *SiTEF*, followed a comparable trend (Fig. 2e). In addition, the amount of the phytosterol stigmasta‐5,22‐dien‐3β‐ol (hereafter stigmasterol) increased upon colonization with *S. indica* and over time, while the amount of 24β‐ethylcholest‐5‐en‐3β‐ol (hereafter β‐sitosterol) remained unchanged (Fig. S4A,B). Thus, the ratio of stigmasterol to β‐sitosterol increased in *S. indica‐*colonized roots at 14 dpi (Fig. S4C). Stigmasterol, which is synthesized by desaturation of β‐sitosterol, has been shown to accumulate in several plant–microbe interactions (Griebel & Zeier, 2010; Wang *et al*., 2012; Huang *et al*., 2022). These data demonstrate that the plant modifies its phytosterol content in response to *S. indica* colonization.

Having detected immunogenic ergosterol in colonized roots (Fig. 2c,d), we next analyzed the lipid content of apoplastic fluid of *S. indica*‐colonized and mock‐treated barley roots. Ergosterol was detected in the apoplastic fluid of colonized roots but was absent in mock‐inoculated samples (Fig. 2f,g), demonstrating its relevance during the *S. indica*–barley interaction.

### Ergosterol pretreatment desensitizes barley roots to fungal lipid‐induced immune responses

**Fig. 3:**
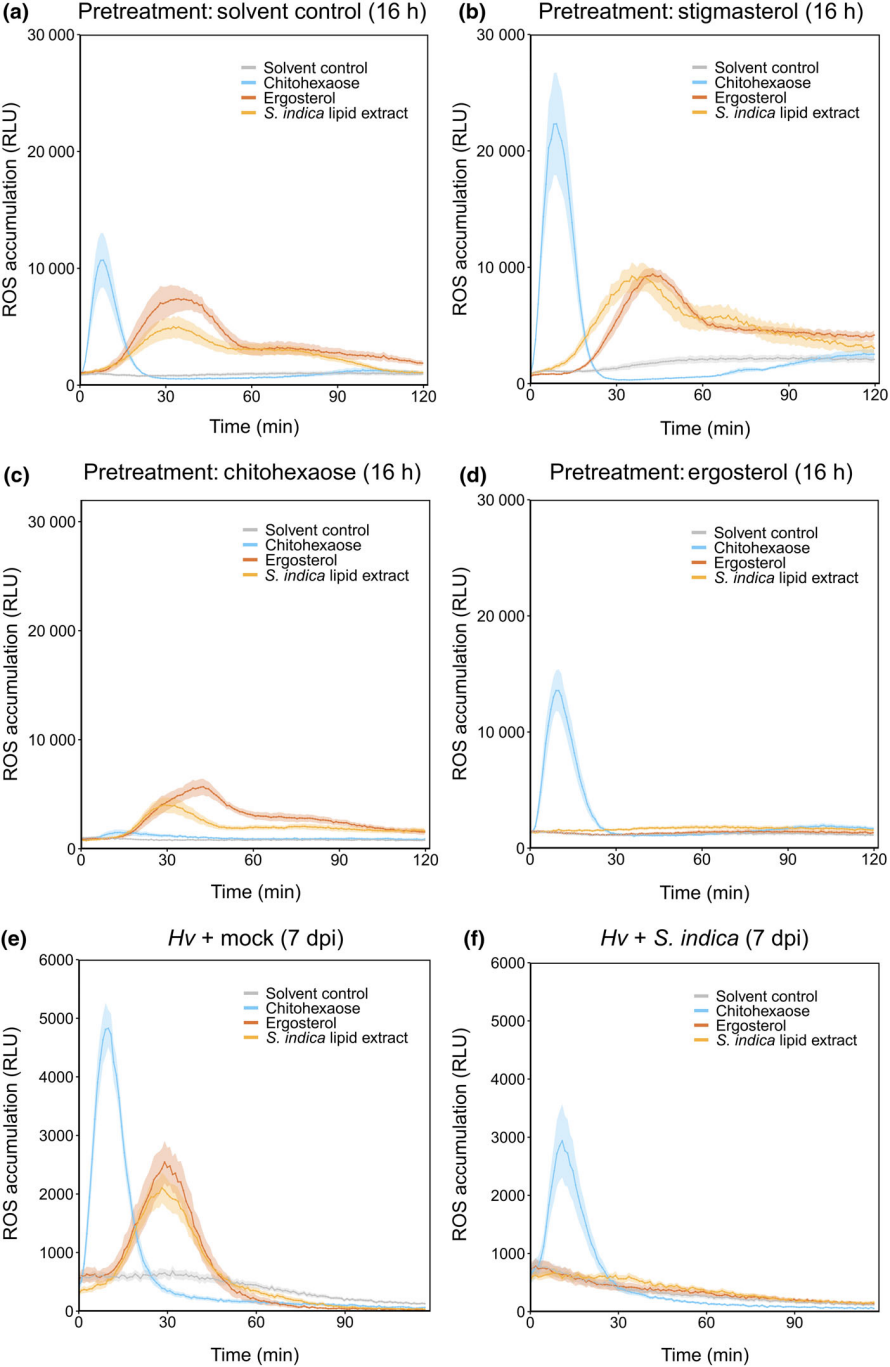
Ergosterol pretreatment and *Serendipita indica* colonization prevents lipid‐induced ROS accumulation. (a–d) Reactive oxygen species (ROS) accumulation in roots of 7‐d‐old barley plants, treated with the indicated elicitors or solvent control after pretreatment with solvent control (a), stigmasterol (b), chitohexaose, (c) or ergosterol (d). (e, f) ROS accumulation in mock‐treated (e) or *S. indica*‐colonized barley roots (f) at 7 d post inoculation (dpi). Roots were washed thoroughly before assay preparations to remove extraradical fungal hyphae. Values represent means ± SEM from eight wells, each containing three root pieces. The following concentrations and dilutions were used ‐ treatments: chitohexaose and ergosterol, 250 nM; *S. indica* lipid extract, 1 : 160 (v/v) dilution; and pretreatments: 5 μM each. All pretreatment and treatment solutions contained a final amount of 1 : 40 (v/v) methanol. *Hv*, *Hordeum vulgare* (barley); RLU, relative luminescence unit; ROS, reactive oxygen species.

MAMP perception triggers complex signaling cascades in plants, including the internalization and degradation of receptor complexes. This process, known as receptor‐mediated endocytosis, serves as a regulatory mechanism to prevent constitutive activation of immune responses (Robatzek *et al*., 2006; Claus *et al*., 2018). Simultaneously, it may trigger *de novo* biosynthesis of ligand‐free receptors to replace the activated ligand‐bound receptors, thereby enabling resensitization (Smith *et al*., 2014). Consequently, subsequent exposure to the same MAMP or MAMPs that share receptor complexes results in a diminished or completely abolished immune response. This phenomenon, termed desensitization or refractory period, is crucial for maintaining cellular homeostasis and preventing excessive energy expenditure on continuous immune activation. Subsequent exposure experiments can be used to distinguish whether different MAMPs share the same receptor and signaling components. Here, barley roots were either pretreated with a solvent control, stigmasterol, chitohexaose, or ergosterol for 16 h and subsequently treated with solvent control, chitohexaose, ergosterol, or *S. indica* lipids. Pretreatment with solvent control or stigmasterol affected neither the chitohexaose‐induced ROS burst nor the ergosterol‐ or *S. indica* lipid‐induced ROS burst (Fig. 3a‐b). In chitohexaose pretreated roots, the chitohexaose‐induced ROS burst was abolished, whereas ergosterol and *S. indica* lipids induced a ROS burst similar to ergosterol and *S. indica* lipid treatment of solvent control‐ or stigmasterol pretreated roots (Fig. 3c). The opposite was observed in ergosterol pretreated roots. Here, no ROS burst was elicited by ergosterol and *S. indica* lipids, whereas chitohexaose treatment induced ROS accumulation (Fig. 3d). These data suggest that ergosterol and *S. indica* lipids share common signaling components but are perceived by a distinct receptor from chitohexaose.

Distinct receptor complexes were shown to recruit similar co‐receptor kinases such as the receptor‐like kinase brassinosteroid‐insensitive 1‐associated kinase 1 (BAK1)/ somatic embryogenesis receptor kinase 3 (SERK3), a co‐receptor involved in several PTI signaling pathways, predominantly those associated with leucine‐rich repeat (LRR) receptor‐like kinases (Couto & Zipfel, 2016). To determine whether BAK1/SERK3 is linked to ergosterol perception, we assessed ROS production in leaves of *S. lycopersicum* cv Moneymaker wild‐type (WT) and *serk3a serk3b* mutant plants in response to ergosterol or flg22 as control (Fig. S5). Previously, flg22‐induced ROS burst was shown to be reduced in *S. lycopersicum serk3* mutants (Peng & Kaloshian, 2014). As expected, the flg22‐induced ROS burst was reduced in *serk3a serk3b* mutant leaves compared with WT (Fig. S5A,B). However, the ergosterol‐induced ROS burst was unaltered in *serk3a serk3b* leaves (Fig. S5A,B). This indicates that BAK1/SERK3 is not involved in ergosterol perception in tomato, suggesting that LRR‐type receptor‐like kinases are not involved in ergosterol perception.

Previous studies showed that *S. indica* colonization suppresses ROS bursts induced by flg22 and chitooctaose in *A. thaliana* (Jacobs *et al*., 2011) and by laminarin in barley (Wawra *et al*., 2016). Therefore, we analyzed whether *S. indica* colonization alters the perception of chitohexaose, *S. indica* lipids, and ergosterol in barley (Fig. 3e,f). In *S. indica*‐colonized roots, the peak height of the chitohexaose‐induced ROS burst was only slightly reduced compared to mock‐inoculated roots, whereas the ROS bursts induced by *S. indica* lipids and ergosterol were completely abolished (Fig. 3e,f). These findings suggest that *S. indica* more effectively inhibits the perception of ergosterol and fungal lipids than chitohexaose in barley at this stage of colonization.

### Phosphatidylinositol phosphate metabolic enzymes are phosphorylated upon fungal lipid treatment

**Fig. 4:**
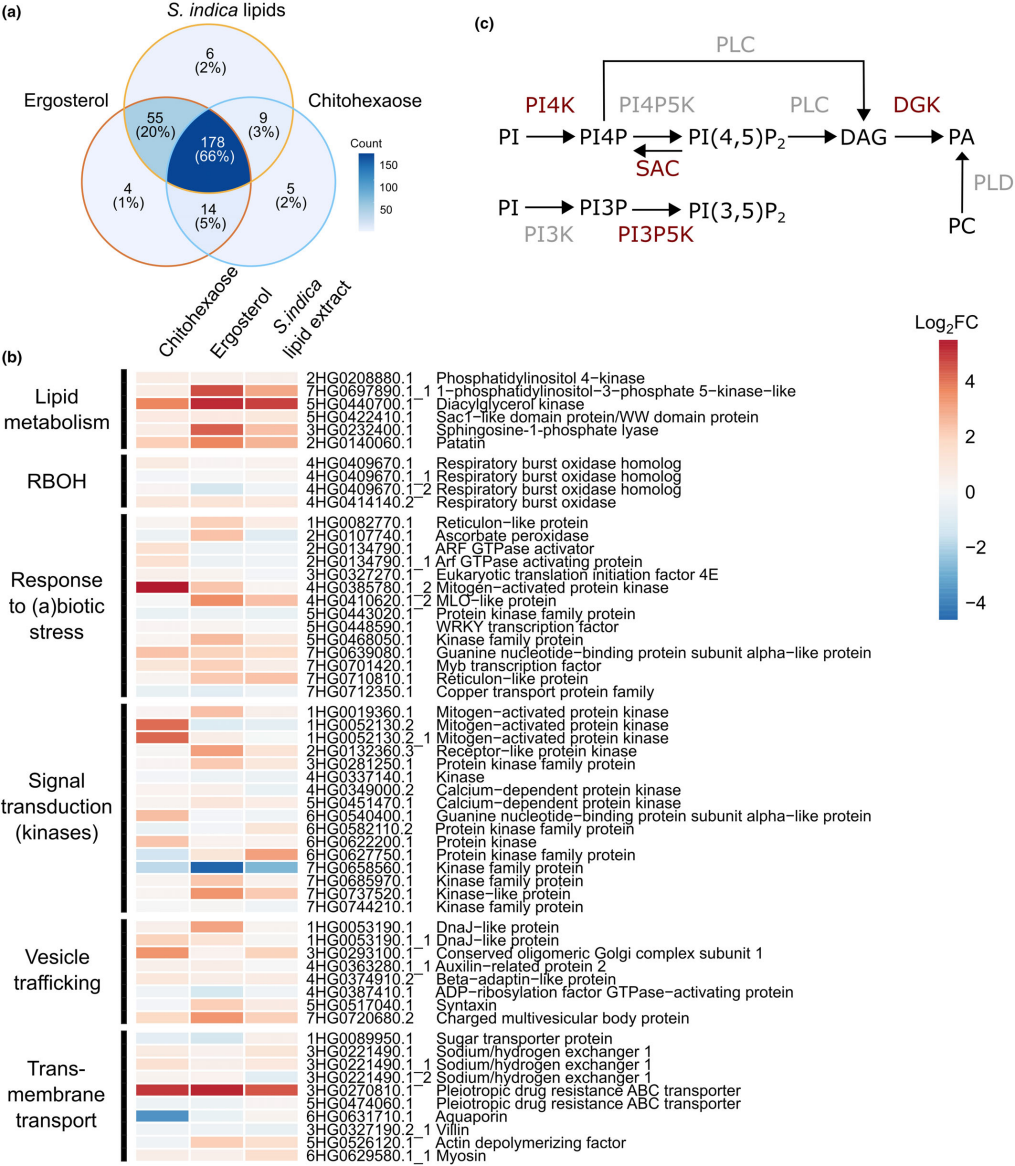
Changes in the phosphoproteome of barley roots upon microbe‐associated molecular patterns (MAMPs) treatment. Phosphorylated peptides were measured using LC‐MS/MS in barley roots in response to chitohexaose, ergosterol, or *Serendipita indica* lipid treatment for 10 min. As negative control, solvent control was used. (a) Venn diagram of phosphorylated peptides found in any of the three treatments but absent from solvent control in unimputed data. (b) Heatmap showing log_2_ fold change (log_2_FC) of imputed intensities of phosphorylated peptides in response to the respective treatments compared with solvent control treatment. Peptides presented here were selected and grouped into functional categories based on Gene Ontology annotations. (c) Schematic depiction of the phosphatidylinositol phosphorylation pathway leading to the production of PA. Enzymes in red were found in phosphoproteome after elicitor treatment as shown in the heatmap. DAG, Diacylglycerol; DGK, Diacylglycerol kinase; PA, Phosphatidic acid; PI, Phosphatidylinositol; PI(3,5)P_2_, Phosphatidylinositol‐3,5‐diphosphate; PI(4,5)P_2_, Phosphatidylinositol‐4,5‐diphosphate; PI3K, Phosphatidylinositol‐3 kinase; PI3P, Phosphatidylinositol‐3‐phosphate; PI3P5K, Phosphatidylinositol‐3‐phosphate‐5 kinase; PI4K, Phosphatidylinositol‐4 kinase; PI4P, Phosphatidylinositol‐4‐phosphate; PI4P5K, Phosphatidylinositol‐4‐phosphate‐5‐kinase; PLC, Phospholipase C; PLD, Phospholipase D; RBOH, Respiratory burst oxidase homolog; SAC, Sac1‐like domain containing protein (PI(4,5)P_2_ phosphatase).

Phosphorylation plays a pivotal role in immunity signaling as one of the primary post‐translational modifications used to rapidly propagate signals within cells. This reversible process allows for quick and dynamic responses to external stimuli. To elucidate the early signal transduction events triggered by fungal lipids and identify key proteins involved in the initial steps of the signaling cascade activated by different elicitors, we conducted a comprehensive analysis of the phosphoproteome in barley roots. Samples were treated for 10 min with ergosterol, *S. indica* lipid extract, chitohexaose, or solvent control as a negative control (Fig. 4). A total of 4502 phosphorylated peptides were detected across all treatments (Table S1A). Of those, 271 were absent from the solvent control treatment but present in at least one of the three elicitor treatments in the unimputed data, and therefore contained candidates for proteins involved in MAMP‐mediated signal transduction (Table S1B). Based on Gene Ontology (GO) terms, candidate proteins were selected and grouped into functional categories (Table S1C). 66% of the treatment‐specific phosphorylated peptides were shared between all three treatments, indicating a conserved response in phosphorylation in response to chitohexaose, ergosterol, and fungal lipids, while 20% were shared between ergosterol and *S. indica* lipid treatment alone (Fig. 4a). A large portion of the phosphorylated candidate peptides refer to proteins that are predicted to be involved in responses to (a)biotic stress or signal transduction, such as MAPK, calcium‐dependent protein kinases, and WRKY transcription factors. Interestingly, a Mildew resistance locus O (MLO)‐like protein was phosphorylated specifically upon lipid treatment (Fig. 4b; Table S1B). MLO proteins were first identified in barley, where their mutations confer resistance against the pathogen *Blumeria graminis* f. sp. *hordei* (Piffanelli *et al*., 2002) and recently also shown to be involved in *S. indica* colonization (Hilbert *et al*., 2020). We further detected proteins potentially involved in vesicle trafficking and transmembrane transport, processes known to be important in PTI (Fig. 4b; Table S1C). Moreover, we identified phosphorylation of proteins, which are predicted to be involved in lipid metabolism and function in plant defense responses, namely a patatin and a sphingosine‐1‐phosphate lyase (Fig. 4b). Patatins have lipid acyl hydrolase function (Rydel *et al*., 2003) and patatin‐like phospholipase As are involved in phospholipid signaling (Ryu, 2004; Canonne *et al*., 2011) and pathogen response (La Camera *et al*., 2005; Kim *et al*., 2014; Jang *et al*., 2020). Sphingosine‐1‐phosphate lyases are involved in sphingosine‐1‐phosphate metabolism, an important signaling molecule in the regulation of drought stress, plant disease resistance, and programmed cell death (Michaelson *et al*., 2016; Zhang *et al*., 2014; Seo *et al*., 2021). Additionally, a phosphatidylinositol 4‐kinase (PI4K), 1‐phosphatidylinositol‐3‐phosphate 5‐kinase‐like (PI3P5K), diacylglycerol kinase (DGK), and Sac1‐like domain containing protein, four proteins potentially involved in PIP‐mediated signaling were phosphorylated (Fig. 4b). PI4K and PI3P5K can phosphorylate PI and PI3P, respectively, producing PI4P and PI(3,5)P_2_. PI4P and PI(4,5)P_2_ can be substrates for phospholipase C (PLC) to produce diacylglycerol (DAG), which again can be phosphorylated by DGKs to produce PA (Fig. 4c). In addition, PA can also be produced from unspecific hydrolysis of all phospholipids by phospholipase D (PLD) (Fig. 4c). The Sac1‐like or suppressor of actin (SAC) domain containing family of proteins are phosphoinositide phosphatases, which dephosphorylate PIPs and therefore are involved in PIP turnover (Mao & Tan, 2021) (Fig. 4c). Taken together, these findings suggest a rapid activation of PIP signaling in response to fungal elicitors as part of the barley root immune response.

### PA enhances the ergosterol‐induced ROS burst

**Fig. 5:**
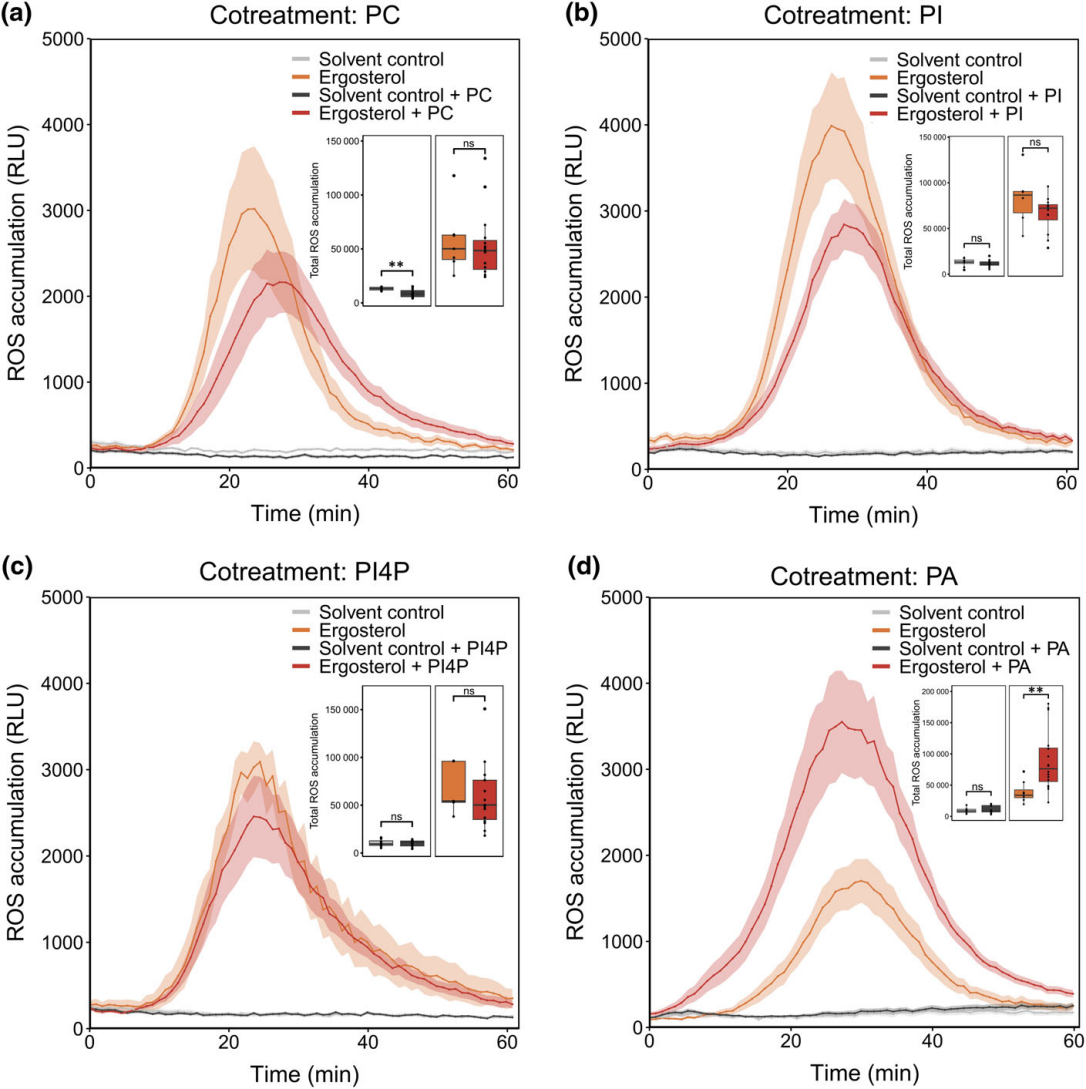
Phosphatidic acid (PA) cotreatment enhances the ergosterol‐induced reactive oxygen species (ROS) burst. (a–d) ROS accumulation in roots of 4‐d‐old barley plants, treated with the indicated elicitors or solvent control as negative control. Values represent means ± SEM from eight (without cotreatment) or 16 (with cotreatment) wells, each containing three root pieces. Ergosterol and all phospholipids (PC, PI, PI4P, PA) were used at a final concentration of 250 nM. All treatments contained a final amount of 1 : 40 (v/v) methanol. Insets: total ROS accumulation depicted as boxplots from the same data. Boxplots depict the interquartile range (IQR) ranging from the lower quartile Q1 (25^th^ percentile) to the upper quartile Q3 (75^th^ percentile). The horizontal line inside the box depicts the median. Data points outside 1.5 × IQR are depicted as outliers (thicker black dots). Asterisks indicate significant differences based on Student's *t*‐test: ns, not significant; **, *P* ≤ 0.01 . PA, phosphatidic acid; PC, phosphatidylcholine; PI, phosphatidylinositol; PI4P, phosphatidylinositol‐4‐phosphate; RLU, relative luminescence unit; ROS, reactive oxygen species.

DGK phosphorylation suggests that barley might synthesize PA from DAG in response to ergosterol and *S. indica* lipid treatment. PLC‐DGK‐derived PA was recently shown to enhance the MAMP‐induced ROS burst in *A. thaliana* (Zhang *et al*., 2009; Kalachova *et al*., 2022; Kong *et al*., 2024; Qi *et al*., 2024). Apoplastic ROS is primarily produced by NADPH‐oxidases, also known as respiratory burst oxidase homologs (RBOHs), which are activated through phosphorylation. In our phosphoproteome analysis, we detected phosphorylation of HvRBOHI3 (4HG0409670.1) and HvRBOHB1 (4HG0414140.2) – annotation based on (Mahalingam *et al*., 2021) – in samples treated with chitohexaose, ergosterol, or *S. indica* lipid extract (Fig. 4b). We therefore tested the influence of PA, PI, and PI4P on the chitohexaose‐ and ergosterol‐induced ROS bursts in barley roots. As an additional control, we used the phospholipid phosphatidylcholine (PC). Addition of PC, PI, or PI4P did not affect the chitohexaose‐ or ergosterol‐induced ROS burst in barley roots (Figs 5a–c, S6A–C). By contrast, cotreatment with PA significantly enhanced the ergosterol‐induced ROS burst, resulting in both higher peak maxima and an overall increase in total ROS production (Fig. 5d). Furthermore, cotreatment with PA liposomes, which are believed to facilitate uptake of PA, yielded similar results (Fig. S7). Conversely, addition of PA did not enhance chitohexaose‐induced ROS production in barley roots (Fig. S6D). To exclude a chitohexaose concentration‐dependent effect, we tested a range of chitohexaose concentrations and confirmed that PA did not enhance the chitohexaose‐induced ROS burst across all tested concentrations (Fig. S8). These results reveal a differential impact of phospholipids on MAMP‐induced ROS production in barley, with PA specifically enhancing the ergosterol‐triggered ROS burst but not the chitohexaose‐induced response, further suggesting distinct signaling pathways for these MAMPs.

### Diterpene biosynthesis is activated upon fungal lipid treatment

**Fig. 6:**
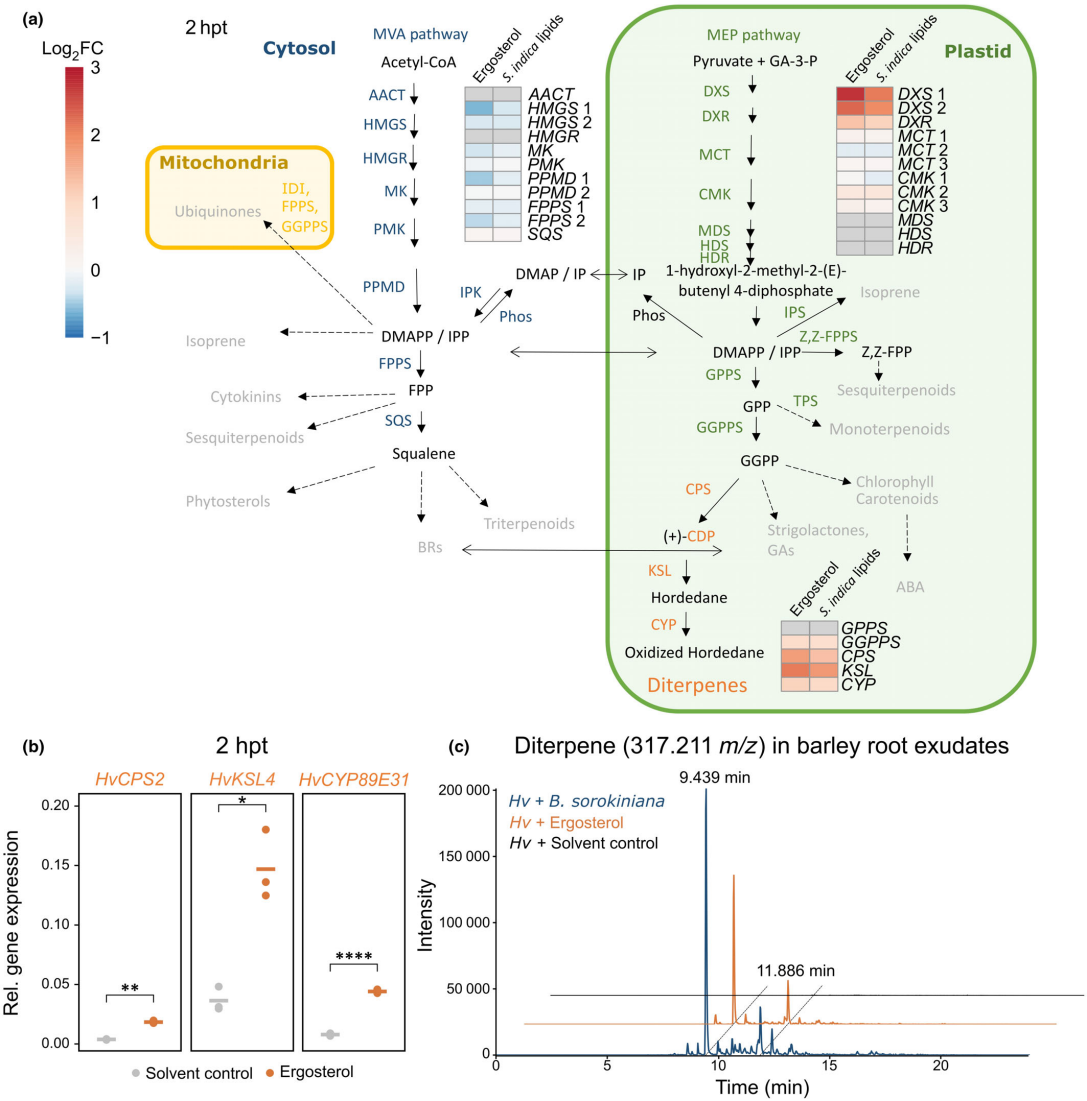
Fungal lipid treatment leads to induction of the 2‐C‐methylerythritol 4‐phosphate (MEP) pathway and exudation of diterpenes in barley roots. (a) Schematic representation of the plants MEP and mevalonate (MVA) pathway (adapted from Liao *et al*. (2016), with permission from Elsevier). Boxes indicate relative expression (Log2FC) of the respective barley genes upon ergosterol or *Serendipita indica* lipid treatment relative to solvent control treatment. Expression was measured in an RNA‐Seq analysis of barley, treated for 2 h with the respective elicitors. Ergosterol was used at a final concentration of 250 nM and *S. indica* lipid extract at a final dilution of 1 : 160 (v/v). Gray boxes, NA. Dashed arrows indicate multiple reaction steps. (b) Confirmation of induction of diterpene biosynthesis genes *HvKSL4*, *HvCPS2* and *HvCYP89E31* in barley roots after treatment with ergosterol (250 nM) for 2 h. Gene expression was determined with quantitative reverse transcription polymerase chain reaction, relative to the housekeeping gene *HvUBI*. Horizontal lines depict mean values. Asterisks indicate significant differences based on Student's *t*‐test: *, *P* ≤ 0.05; **, *P* ≤ 0.01; ****, *P* ≤ 0.0001. (c) LC‐MS/MS chromatogram of one of the major diterpenes found in root exudates of barley plants treated with solvent control, ergosterol (250 nM) or colonized by *Bipolaris sorokiniana* (positive control) for 6 d. The experiment was repeated with similar results. Ergosterol and solvent control treatments contained a final amount of 1 : 40 (v/v) methanol. CPS, *ent‐*copalyl diphosphate synthase; CYP, cytochrome P450 oxygenase; DXR, 1‐deoxy‐xylulose 5‐phosphate reductoisomerase; DXS, 1‐deoxy‐ xylulose 5‐phosphate synthase; hpt, hours post treatment; *Hv*, *Hordeum vulgare* (barley); KSL, kaurene synthase like.

In addition to changes in the phosphoproteome, we investigated transcriptomic changes upon treatment with ergosterol or *S. indica* lipids at 2 hpt to identify candidate genes involved in downstream responses. The analysis revealed that 244 genes were significantly differentially expressed upon ergosterol or *S. indica* lipid treatment (log2FC ≥ 1, ≤ −1; *q*‐value ≤ 0.05) (Table S2A). Among the most upregulated genes were two genes encoding 1‐deoxy‐xylulose5‐phosphate synthases (*DXS*) and one gene encoding a 1‐deoxy‐d‐xylulose‐5‐phosphate reductoisomerase (*DXR*), the enzymes that perform the committing steps of the 2‐C‐methylerythritol 4‐phosphate (MEP or nonmevalonate) pathway (Table S2; Fig. 6a). In addition, expression of multiple genes, potentially involved in the MEP pathway was induced, whereas expression of genes potentially involved in the mevalonate (MVA) pathway was downregulated or not detected at all (Fig. 6a). The MEP pathway leads to the production of isoprenoid intermediates, which can be converted to hordedanes, a class of diterpene phytoalexins produced in barley in response to fungal treatment (Fig. 6a; Liu *et al*., 2024). Indicative of an induction of diterpene synthesis, we found homologs of geranylgeranyl diphosphate synthase (*GGPPS*), *ent*‐copalyl diphosphate synthase 2 (*CPS2*), kaurene synthase‐like 4 (*KSL4*), and cytochrome P450 oxygenases (*CYPs*) among the strongest induced genes upon fungal lipid treatment (Fig. 6a; Table S2). We verified the induction of *HvCPS2*, *HvKSL4*, and *HvCYP89E31* upon the addition of ergosterol via quantitative reverse transcription polymerase chain reaction (Fig. 6b).

To investigate whether diterpenes are synthesized in response to ergosterol treatment, we analyzed root exudates of barley roots treated with either ergosterol or roots infected by *B. sorokiniana* as positive control, by LC‐MS. We detected two major diterpenes with mass‐to‐charge ratio (*m/z*) of 315.196 and 317.211 – previously identified in root exudates of *B. sorokiniana*‐colonized roots (Liu *et al*., 2024) – in both root exudates of ergosterol‐treated and *B. sorokiniana*‐colonized barley roots (Figs 6c, S9). These findings confirm that ergosterol perception by barley triggers the biosynthesis and exudation of antimicrobial phytoalexin diterpenes.

## Discussion

### *Serendipita indica* colonization modulates pattern‐triggered immunity during fungal lipid perception

In this study we demonstrate that ergosterol and total lipids isolated from the beneficial root endophyte *S. indica* induce hallmarks of PTI in barley (Figs 1, S1, S2). Apoplastic ROS production and increase in [Ca^2+^]_cyt_ are recognized as two of the earliest and often interlinked signaling mechanisms activated in plants in response to MAMP perception (Ranf *et al*., 2011; Qi *et al*., 2017; Marcec *et al*., 2019). In addition to the Ca^2+^‐independent activation of *A. thaliana* RBOHD by phosphorylation via the cytosolic kinase BIK1 (Kadota *et al*., 2015), intracellular Ca^2+^ was shown to activate *A. thaliana* RBOHD through direct binding of Ca^2+^ to EF hand motifs (Ogasawara *et al*., 2008; Kimura *et al*., 2012) and phosphorylation by Ca^2+^‐dependent protein kinases (Dubiella *et al*., 2013; Seybold *et al*., 2014). In addition, increase in [Ca^2+^]_cyt_ is required for initiation of the flg22‐induced ROS burst in *A. thaliana* (Marcec & Tanaka, 2022). In our experiments, chitohexaose treatment induced both a strong Ca^2+^‐ and ROS burst in barley roots (Fig. 1c,e) as previously reported (Chandrasekar *et al*., 2022). By contrast, ergosterol and *S. indica* lipids induced no or only minor increase in [Ca^2+^]_cyt_ but a distinct ROS burst in barley roots (Fig. 1c,e), suggesting that Ca^2+^‐influx might not be required for the lipid‐induced ROS burst in barley roots.

Given that plants encode various RBOH isoforms with distinct functions (Suzuki *et al*., 2011; Zhang *et al*., 2023), the perception of different MAMPs in barley may activate specific RBOH isoforms, some of which might not require Ca^2+^ for activation (Mahalingam *et al*., 2021). Phosphoproteome analysis of barley roots revealed phosphorylation of *Hv*RBOHB1 (4HG0414140.2) and *Hv*RBOHI3 (4HG0409670.1), indicating they might be involved in MAMP perception in barley. Further identification and characterization of the barley RBOHs is needed to clarify their specific functions in fungal lipid perception and MAMP‐induced responses.

Desensitization of MAMP‐induced immunity in barley revealed that barley roots employ distinct receptors for chitohexaose and ergosterol perception (Fig. 3a–d). Similarly, membrane hyperpolarization induced by ergosterol in *Mimosa pudica* cells was abolished upon subsequent exposure with ergosterol but not chitosan or cholesterol (Amborabé *et al*., 2003). Further evidence in tomato indicates that BAK1/SERK3 is not involved in ergosterol recognition (Fig. S5). Moreover, colonization by *S. indica* more effectively suppressed the perception of ergosterol compared with chitohexaose perception (Fig. 3e,f) and the activation of early immune responses was delayed with fungal lipids compared with chitohexaose treatment (Figs 1, S1).

The distinct perception of ergosterol compared with other MAMPs may be attributed to their differing physicochemical properties. Unlike water‐soluble MAMPs such as flg22 and chitohexaose, ergosterol is hydrophobic and poorly soluble in water. Ergosterol is present in the membrane of fungal extracellular vesicles (EVs; Rodrigues, 2018) and animal pathogens such as *Cryptococcus neoformans* or *Candida albicans* were shown to secrete ergosterol‐containing EVs to transport macromolecules across the cell wall (Rodrigues *et al*., 2007; Vargas *et al*., 2015). The amount of ergosterol in liposomes correlated with the ability to induce pyroptosis (Wellington *et al*., 2014; Koselny *et al*., 2018). Plant colonizing fungi can also release EVs into the apoplast to secrete for instance small‐interfering RNAs or effector proteins (An *et al*., 2006; Lo Presti & Kahmann, 2017; Rutter & Innes, 2018; Garcia‐Ceron *et al*., 2021; Li *et al*., 2022; Oliveira‐Garcia *et al*., 2023). Recently, it was reported that ergosterol‐induced nanodomains are required for the activation of clathrin‐mediated endocytosis to translocate fungal effectors into the host during the *Magnaporthe oryzae*–rice interaction (Guo *et al*., 2023; Oliveira‐Garcia *et al*., 2023).

Thus, upon plant colonization, ergosterol could be released into the apoplast via EVs, making it accessible for perception by the plant. Ergosterol is able to integrate into membranes, potentially inducing the formation of nanodomains that significantly alter the structure and fluidity of the membrane (Xu *et al*., 2001; Klemptner *et al*., 2014). In plant plasma membranes, such perturbations could activate membrane‐embedded mechanosensors or other receptors sensitive to structural changes in the lipid bilayer, as observed with the lipopeptide surfactin from root‐associated rhizobacteria (Pršić *et al*., 2023).

This complex process could explain the delayed ROS burst observed with ergosterol compared to chitohexaose perception. The time required for membrane integration and subsequent signaling cascades may exceed that of direct recognition by apoplastic receptors, accounting for the differences in timing of the immune response between these MAMPs. Still perception via direct binding to a membrane receptor is not excluded.

*Serendipita indica* colonization led to an increase in stigmasterol in barley roots (Fig. S4). Stigmasterol is synthesized via C22 desaturation of β‐sitosterol (Aboobucker & Suza, 2019) and was shown to have a higher ordering effect on the membrane than β‐sitosterol (Grosjean *et al*., 2015). Changes in the stigmasterol‐to‐β‐sitosterol content affect microbial colonization and nutrient exchange, probably by influencing membrane rigidity (Griebel & Zeier, 2010; Wang *et al*., 2012; Huang *et al*., 2022). Furthermore, sterols regulate the spatial distribution of membrane lipids, for example PIPs and certain proteins, such as transporters, RBOHs (Der *et al*., 2024) or callose‐modifying enzymes (Grison *et al*., 2015), potentially modulating plant immune responses or receptor availability. Therefore, the increase in stigmasterol during *S. indica* colonization could lead to rigidified membranes and modified spatial distribution of membrane proteins, altering nutrient transport, fungal penetration, and immunity activation.

### Fungal lipid treatment induces phosphorylation of proteins and expression of genes involved in plant lipid signaling

Next to canonical signaling components such as MAPK and receptor‐like cytoplasmic kinases, we found PI4K, PI3P5K, DGK, and a Sac1‐like domain containing protein to be phospho‐regulated upon fungal lipid treatment (Fig. 4). These enzymes could be involved in the synthesis and homeostasis of the plant lipids PI4P, PI(3,5)P_2_ and PA. In *A. thaliana*, *At*PI4Kα1 (At1g49340), *At*FAB1A (At4g33240), and *At*PLC2 (At3g08510), involved in PIP signaling, were detected in the plasma membrane‐associated proteome after ergosterol treatment (Khoza *et al*., 2019).

Barley PI4K (2HG0208880.1, Fig. 4b) shows highest sequence similarity to *A. thaliana* PI4Kγ7 (At2g03890), and wheat PI4Kγ6‐like (XP_044459596), which was shown to be involved in salt‐ and drought stress tolerance (Liu *et al*., 2013) and barley PI3P5K (7HG0697890.1, Fig. 4b) is most similar to *A. thaliana* formation of aploid and binucleate cells1 A (FAB1A, At4g33240) and FAB1B (At3g14270), involved in endosome maturation and microtubule association, controlling protein trafficking (Hirano *et al*., 2011, 2015). While PI4K and PI3P5K phosphorylate PIPs, the barley Sac1‐like domain containing protein (5HG0422410.1, Fig. 4b) is the homolog of Arabidopsis SAC9, a plant‐specific PI(4,5)P_2_‐5 phosphatase that likely dephosphorylates PI(4,5)P_2_ to PI4P (Williams *et al*., 2005; Lebecq *et al*., 2022). The interplay of PIP kinases and the Sac1‐like protein in barley likely regulates the formation of membrane regions enriched in PI4P and PI(4,5)P_2_. These could serve as signaling hubs for membrane receptors (Antignani *et al*., 2015) involved in plant immunity during colonization of barley with *S. indica* and control vesicle trafficking (Synek *et al*., 2021) and fungal accommodation (Ivanov & Harrison, 2019; Qin *et al*., 2020).

In addition, DGK phosphorylation suggests that barley might synthesize PA from DAG in response to ergosterol and *S. indica* lipid treatment. Barley DGK (5HG0440700.1, Fig. 4b) showed highest sequence similarity to *A. thaliana* DGK1 and 2 (At5g07920, At5g63770), which were shown to be involved in cold stress tolerance in *A. thaliana* (Arisz *et al*., 2013). PLC and PA were described as important modulators of ROS levels under biotic stress (D'Ambrosio *et al*., 2017; Seth *et al*., 2024), and PA was shown to accumulate in a burst‐like fashion, similar to ROS, in response to microbial infection (Andersson *et al*., 2006; Zhang & Xiao, 2015). Moreover, earlier studies demonstrated ROS production upon PA treatment of tobacco cells, *A. thaliana* guard cells or PA infiltration in *A. thaliana* leaves (Sang *et al*., 2001; De Jong *et al*., 2004; Park *et al*., 2004; Zhang *et al*., 2009). PA interacts with many different proteins in *A. thaliana*, targeting them to membranes and activating or inhibiting their enzymatic functions (Testerink & Munnik, 2005; Pokotylo *et al*., 2018; Yao & Xue, 2018). Thus, PA can regulate the availability of receptors and signaling components involved in MAMP perception, as recently demonstrated for *A. thaliana* RBOHD. PLC2‐DGK5β‐derived PA inhibited vacuolar degradation of RBOHD in response to flg22 and chitin treatment, thereby enhancing RBOHD‐mediated ROS production in *A. thaliana* leaves (D'Ambrosio *et al*., 2017; Kong *et al*., 2024; Qi *et al*., 2024). Cotreatment of barley roots with PA enhanced the ergosterol‐induced ROS burst (Fig. 5d), indicating PA might stabilize RBOH in barley roots in response to MAMP treatment as well. However, we did not observe an enhanced ROS burst upon chitohexaose treatment with PA cotreatment in barley roots or a ROS burst triggered by PA alone (Figs 5, S6, S8). PA synthesis was shown to be activated by *S. indica* colonization and involved in *S. indica* mediated growth promotion of *A. thaliana*, via a pathway involving a PLD, 3‐phosphoinositide‐dependent protein kinase1 (PDK1), and oxidative signal inducible 1 (OXI1) cascade (Camehl *et al*., 2011). Expression of *PDK1*, which is activated by PA (Anthony *et al*., 2004), and *OXI1*, which is necessary for ROS burst‐mediated signaling in *A. thaliana* (Rentel *et al*., 2004), is upregulated during *S. indica* colonization, supporting a connection between PA and ROS signaling upon *S. indica* colonization (Camehl *et al*., 2011). It remains unknown how *S. indica* colonization suppresses the lipid‐induced ROS burst (Fig. 3f) but since PA enhances the ergosterol‐induced ROS burst (Fig. 5d), modulating PA synthesis could be one way to also modulate lipid‐induced host immunity. However, since addition of PA is not required for the ergosterol‐induced ROS burst, other pathways are likely also targets of *S. indica* for suppression of host immunity.

Additionally, we have observed that the perception of *S. indica* lipids and ergosterol induces the expression of the diterpene biosynthesis genes, *HvCPS2*, *HvKSL4*, and *HvCYP89E31* (Fig. 6a,b). Recently, these genes were shown to be highly induced in response to colonization by the pathogenic fungus *B. sorokiniana* but also slightly by the beneficial root endophyte *Serendipita vermifera*. Diterpenes synthesized by these enzymes have been shown to be exuded by barley roots upon fungal colonization and inhibit germination of several root colonizing fungi including *S. indica* (Sarkar *et al*., 2019; Mahdi *et al*., 2022; Liu *et al*., 2024).

Taken together, these data demonstrate that perception of fungal lipids activates several plant lipid signaling pathways involved in both rapid and downstream immune responses.

### Conclusion

Ergosterol, the predominant sterol in fungal membranes, serves as a MAMP in barley–fungus interactions. During root colonization, *S. indica* hyphae proliferate in the apoplast where ergosterol is present. Although typically confined within fungal membranes, ergosterol could become accessible for plant perception through the release of EVs into the apoplast.

**Fig. 7:**
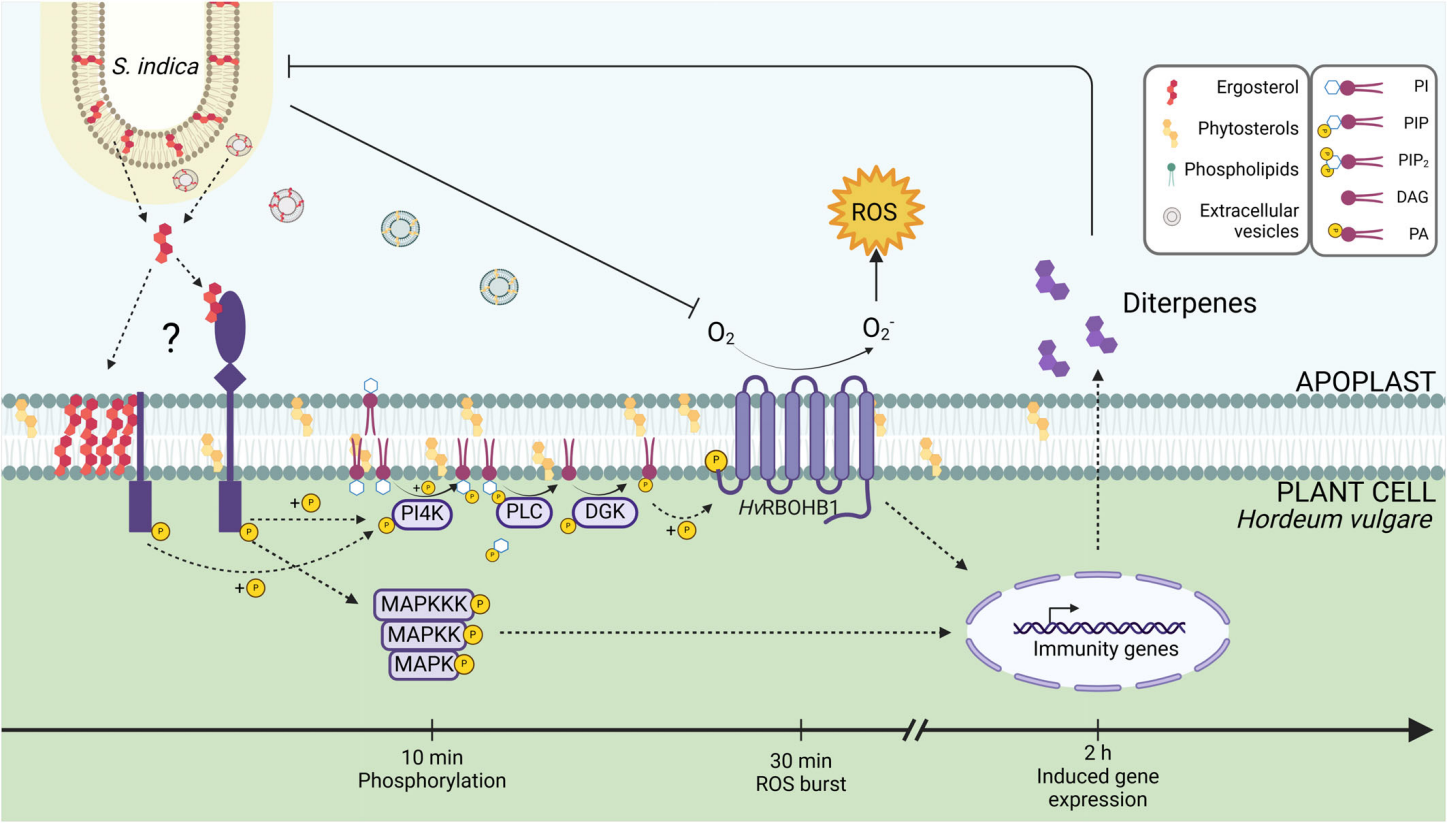
Proposed model of fungal lipid perception in barley roots. We propose two mechanisms for how plants perceive ergosterol. First, ergosterol could directly integrate into the plant plasma membrane, potentially activating membrane‐embedded mechanosensors or specific ergosterol‐binding receptors within the membrane. Second, ergosterol could bind in the apoplast to the extracellular domain of a yet unidentified membrane‐bound receptors (Xu *et al*., 2001; Klemptner *et al*., 2014; Khoza *et al*., 2019; Lindo *et al*., 2020). Upon perception, ergosterol likely initiates a signaling cascade that begins with receptor phosphorylation, leading to phosphorylation of MAPKs (10–30 min) and the rapid activation of PI4K and diacylglycerol kinase (DGK) (10 min). This activation results in the production of phosphatidic acid (PA), which subsequently enhances the activation and stabilization of respiratory burst oxidase homologs (RBOH), amplifying the reactive oxygen species (ROS) burst (30 min). Together, these signaling events lead to increased expression of immunity genes and diterpene biosynthesis genes (2 h), which ultimately results in the exudation of diterpenes, which were shown to inhibit growth of *Serendipita indica*. During colonization, *S. indica* suppresses the ergosterol‐mediated ROS burst. Dashed arrows indicate potential activation, blunt‐ended arrows indicate inhibition. DAG, diacylglycerol; DGK, diacylglycerolkinase; MAPK, mitogen‐associated protein kinase; PA, phosphatidic acid; PI, phosphatidylinositol; PI4K, phosphatidylinositol‐4 kinase; PIP, phosphatidylinositolphosphate; PIP_2_, phosphatidylinositoldiphosphate; PLC, phospholipase C; RBOH, respiratory burst oxidase homolog; ROS, reactive oxygen species. This figure was created using BioRender: https://BioRender.com/v44j538.

Two mechanisms may underlie plant perception of ergosterol. First, ergosterol may bind to the extracellular domain of membrane‐bound receptors in the apoplast. Alternatively, ergosterol could integrate into the plant plasma membrane, subsequently activating membrane‐embedded mechanosensors or other receptors responsive to structural changes in the lipid bilayer. Additionally, the integrated ergosterol may directly interact with specific ergosterol‐binding receptors within the membrane (Fig. 7).

Upon ergosterol perception, a signaling cascade is initiated that leads to the phosphorylation of PI4K, PI3P5K, and DGK, potentially promoting PA synthesis, which in turn, enhances RBOH activation and stabilization, amplifying the ROS burst in barley. Furthermore, phosphoinositides generated from PI4K and PI3P5K contribute to the formation of membrane contact sites that may facilitate vesicle fusion, receptor recruitment, or internalization. This series of signaling events activates MAPK cascades, leading to increased expression of immunity genes and diterpene biosynthesis genes. The culmination of these events results in diterpene exudation, a protective response that deters microbial invaders sensitive to this diterpene class. Notably, *S. indica* has adapted to root colonization and can suppress the fungal–lipid‐induced ROS burst and thus limit diterpene biosynthesis.

Despite ergosterol's important role in plant–microbe interactions and its use as a marker for fungal colonization in crops like barley and corn, it has received limited research attention in recent years. This study provides evidence that fungal–lipid perception in barley roots is mediated through plant–lipid signaling pathways. Further investigation into the specific receptors involved could offer valuable insights into plant–fungal interactions and the mechanisms employed by fungi to overcome plant immune responses.

## Competing interests

None declared.

## Author contributions

PS, MB and AZ designed the research, conceptualized and edited the manuscript. PS, MB and ABE performed the research and analyzed the data. SCS and HN performed the phosphoproteome analysis. PW performed the GC‐TOF‐MS analysis. GUB and AT performed LC‐MS/MS analysis. GH generated the AEQ expressing barley lines. NH and CZ generated the tomato *serk3a serk3b* mutants. All authors were involved in editing the paper. AZ provided funding for the experiments.

## Disclaimer

The New Phytologist Foundation remains neutral with regard to jurisdictional claims in maps and in any institutional affiliations.

## Supporting information


**Fig. S1** Lipids from the beneficial root endophyte *Serendipita indica* induce immunity in barley leaves.
**Fig. S2** Ergosterol induces MAPK phosphorylation in barley roots.
**Fig. S3** *Serendipita indica* lipids are differentially perceived in different plant species.
**Fig. S4** *Serendipita indica* colonization induces modulation of the phytosterol pool in barley.
**Fig. S5** BAK1/SERK3 is not involved in *Serendipita indica* lipid perception in *Solanum lycopersicum* leaves.
**Fig. S6** Phospholipid cotreatment does not enhance the chitohexaose‐induced reactive oxygen species burst in barley roots.
**Fig. S7** Cotreatment with phosphatidic acid liposomes enhances the ergosterol‐induced reactive oxygen species burst in barley roots.
**Fig. S8** Phosphatidic acid cotreatment does not enhance chitohexaose‐induced reactive oxygen species burst in barley roots.
**Fig. S9** Diterpene exudation in response to ergosterol treatment and *Bipolaris sorokiniana* colonization.
**Methods S1** Detailed description of the Materials and Methods section.

**Table S1** Phosphoproteomics data.

**Table S2** RNA‐seq data.

**Table S3** Primers used in this study.
Please note: Wiley is not responsible for the content or functionality of any Supporting Information supplied by the authors. Any queries (other than missing material) should be directed to the *New Phytologist* Central Office.

## Data Availability

The RNA‐Seq data generated in this paper will be available at the National Center for Biotechnology Information (NCBI) Gene Expression Omnibus (GEO), under the GEO accession no. GSE280369 (https://www.ncbi.nlm.nih.gov/geo/query/acc.cgi?acc=GSE280369). The mass spectrometry proteomics data have been deposited to the ProteomeXchange Consortium via the PRIDE partner repository (Perez‐Riverol *et al*., 2022) with the dataset identifier PXD056788 and can be accessed on the PRIDE website. All data supporting the findings of this study are available within the article and Figs S1–S9; Tables S1–S3; Methods S1.
