# Supplementary material for: Ergosterol‐induced immune response in barley involves phosphorylation of phosphatidylinositol phosphate metabolic enzymes and activation of diterpene biosynthesis
Source: New Phytol. 2025 Mar 7;246(3):1236–55. doi: 10.1111/nph.70022 (PMC11982792; doi:10.1111/nph.70022)
Supplement: Supplementary file 1 — Fig. S1 Lipids from the beneficial root endophyte Serendipita indica induce immunity in barley leaves. Fig. S2 Ergosterol induces MAPK phosphorylation in barley roots. Fig. S3 Serendipita indica lipids are differentially perceived in different plant species. Fig. S4 Serendipita indica colonization induces modulation of the phytosterol pool in barley. Fig. S5 BAK1/SERK3 is not involved in Serendipita indica lipid perception in Solanum lycopersicum leaves. Fig. S6 Phospholipid cotreatment does not enhance the chitohexaose‐induced reactive oxygen species burst in barley roots. Fig. S7 Cotreatment with phosphatidic acid liposomes enhances the ergosterol‐induced reactive oxygen species burst in barley roots. Fig. S8 Phosphatidic acid cotreatment does not enhance chitohexaose‐induced reactive oxygen species burst in barley roots. Fig. S9 Diterpene exudation in response to ergosterol treatment and Bipolaris sorokiniana colonization. Methods S1 Detailed description of the Materials and Methods section. [file NPH-246-1236-s003.docx]

## *New Phytologist* Supporting Information

Article title: Ergosterol-Induced Immune Response in Barley Involves Phosphorylation of Phosphatidylinositol Phosphate Metabolic Enzymes and Activation of Diterpene Biosynthesis

Authors: Pia Saake, Mathias Brands, Asmamaw Bidru Endeshaw, Sara Christina Stolze, Philipp Westhoff, Gerd Ulrich Balcke, Götz Hensel, Nicholas Holton, Cyril Zipfel, Alain Tissier, Hirofumi Nakagami, Alga Zuccaro

Article acceptance date: 23 January 2025

The following Supporting Information is available for this article:

**Fig. S1:** Lipids from the beneficial root endophyte *S. indica* induce immunity in barley leaves.

**Fig. S2:** Ergosterol induces MAPK phosphorylation in barley roots.

**Fig. S3:** *S. indica* lipids are differentially perceived in different plant species.

**Fig. S4:** *S. indica* colonization induces modulation of the phytosterol pool in barley.

**Fig. S5:** BAK1/SERK3 is not involved in *S. indica* lipid perception in *S. lycopersicum* leaves.

**Fig. S6:** Phospholipid cotreatment does not enhance the chitohexaose-induced ROS burst in barley roots.

**Fig. S7:** Cotreatment with PA in liposomes enhances the ergosterol-induced ROS burst in barley roots.

**Fig. S8:** PA cotreatment does not enhance chitohexaose-induced ROS burst in barley roots.

**Fig. S9:** Diterpene exudation in response to ergosterol treatment and *B. sorokiniana* colonization.

**Table S1** Phosphoproteomics data

1. Imputed data of all phosphorylated peptides
2. MNAR data of all peptides absent from the control but present in any treatment
3. Data presented in Figure 5B (Selected groups based on GO terms, imputed data)

**Table S2**: RNAseq data

1. Significantly differentially expressed genes
2. Log2FC and tpm of all genes

**Methods S1** Detailed description of materials and methods

**Fig. S1: Lipids from the beneficial root endophyte *S. indica* induce immunity in barley leaves.**

A) Expression of *HvPR10* relative to the housekeeping gene *HvUBI* in barley leaf discs determined by qRT-PCR. Leaf discs were treated with the indicated MAMPs or solvent control for 2 or 24 h. Horizontal lines depict mean values. Letters indicate significant differences based on ANOVA + post hoc Tukey test (p ≤ 0.05). B) ROS accumulation in leaves of seven days old barley plants, treated with the indicated lipid extracts or solvent control as negative control. Values represent means ± SEM from eight wells, each containing one 3 mm leaf disc. All treatments contained a final amount of 1:40 (v/v) methanol. The following concentrations / dilutions were used: *S. indica* lipid extract: 1:160 (v/v), ergosterol: 250 nM, chitohexaose: 25 µM. RLU = Relative luminescence unit; ROS = Reactive oxygen species; hpt = Hours post treatment.


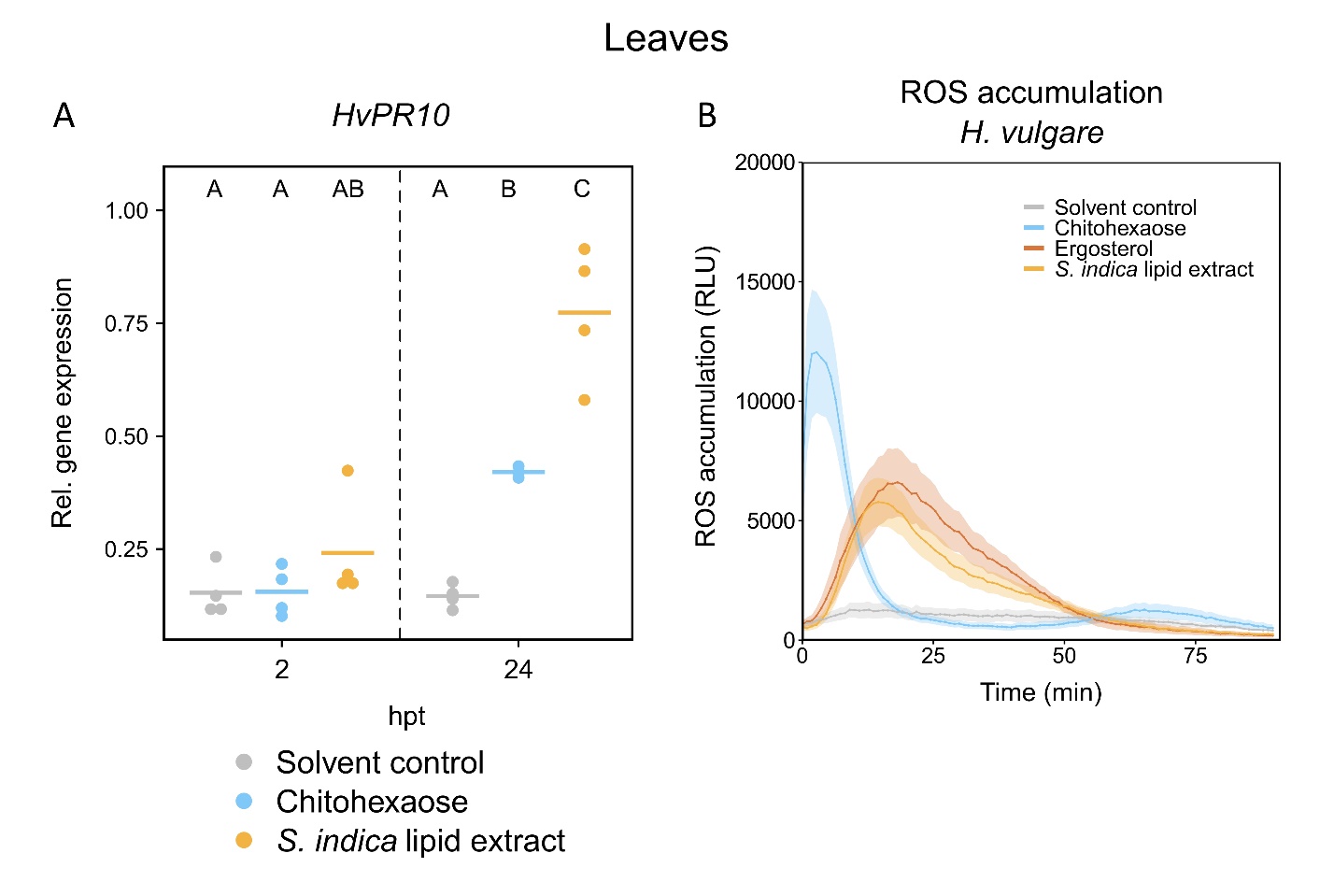


**Fig. S2: Ergosterol induces MAPK phosphorylation in barley roots.**


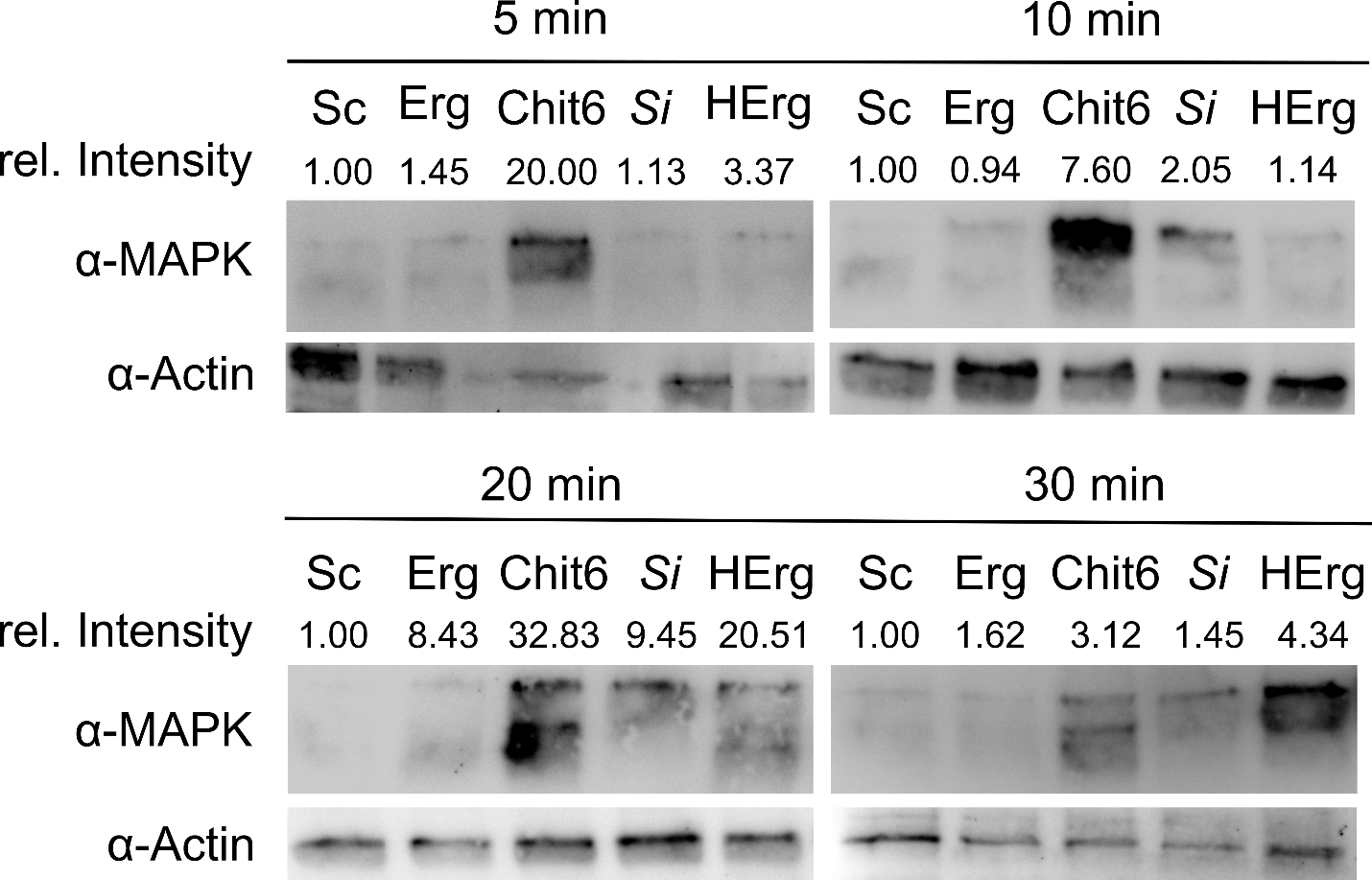
MAPK phosphorylation in roots of seven days old barley plants, treated with the indicated MAMPs or solvent control for 5-, 10-, 20- or 30-min. 1^st^ antibody: Phospho-p44/42 MAPK (upper) or α-Actin as loading control (lower), 2^nd^ antibody: anti-rabbit IgG. Sc = solvent control [1:160], Erg = Ergosterol [250 nM], Chit6 = chitohexaose [250 nM], *Si* = *S. indica* lipid extract [1:160], HErg = Ergosterol [25 µM]. Relative Intensity depicts the MAPK signal intensity normalized to the Actin signal intensity of the same treatment and to the solvent control (Sc) for each timepoint.

**Fig. S3: *S. indica* lipids are differentially perceived in different plant species.**


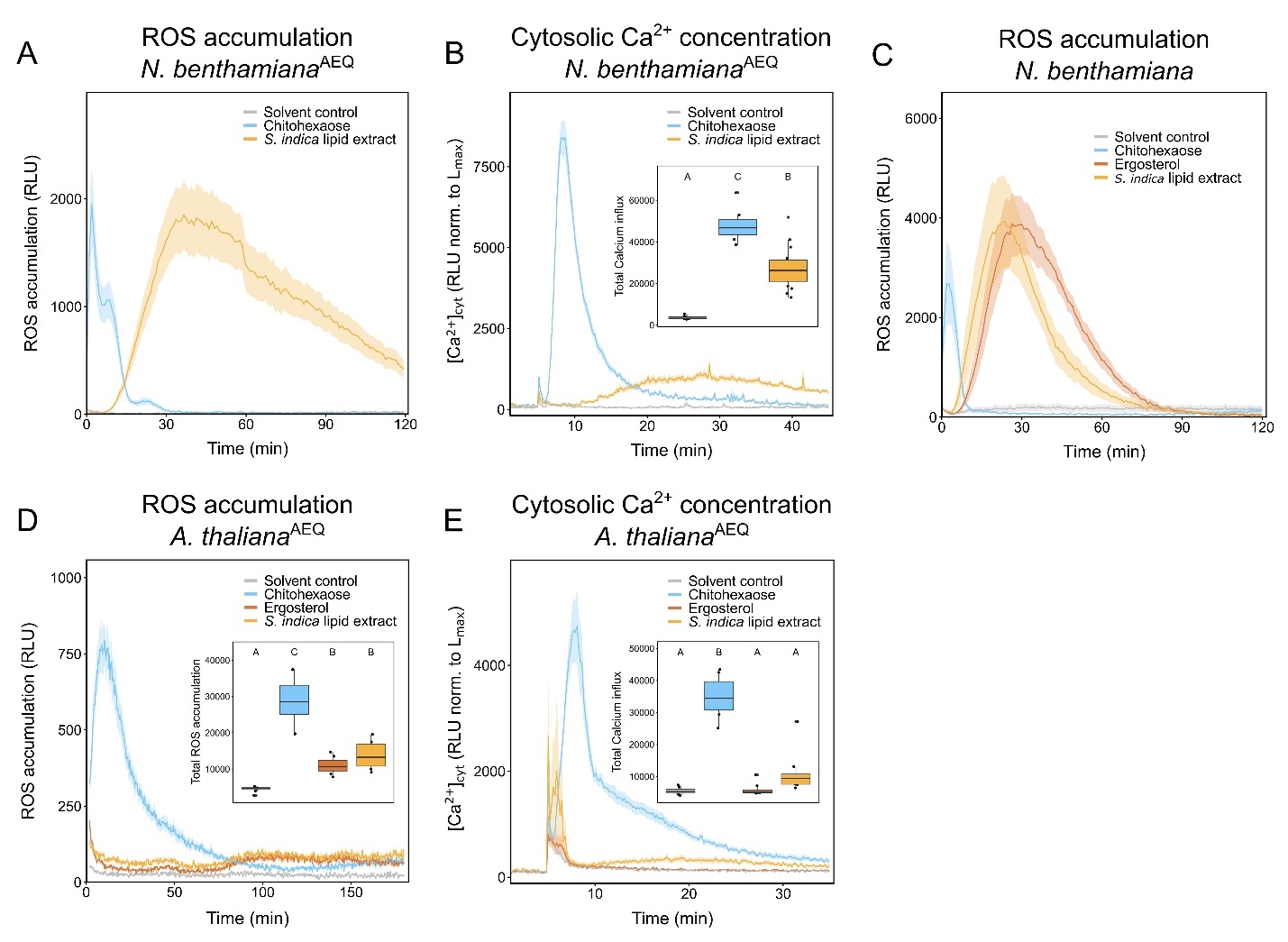
ROS accumulation (A, C, D) and cytosolic Ca^2+^ concentration ([Ca^2+^]_cyt_) (B, E) in leaves of *N. benthamiana*^AEQ^ plants (A-B), leaves of *N. benthamiana* WT plants (C) or *A. thaliana*^AEQ^ seedlings (D-E) treated with the indicated MAMPs or solvent control as negative control. Values represent means (normalized to maximum luminescence (L_max_) over all wells for Ca^2+^) ± SEM from eight wells, each containing one 3 mm leaf disc (A-C) or one seedling (D-E). The following concentrations and dilutions were used: *S. indica* lipid extract: 1:160 (v/v), chitohexaose: 25 µM, ergosterol: 25 µM (D), 250 nM (A, B, C, E). All treatments contained a final amount of 1:40 (v/v) methanol. Insets depict total ROS accumulation from the same experiment. Boxplots depict the interquartile range (IQR) ranging from the lower quartile Q1 (25th percentile) to the upper quartile Q3 (75th percentile). The horizontal line inside the box depicts the median. Data points outside 1.5 x IQR are depicted as outliers (thicker black dots). Letters indicate significant differences based on ANOVA and post hoc Tukey test (p ≤ 0.05). RLU = Relative luminescence unit; ROS = Reactive oxygen species.

**Fig. S4: *S. indica* colonization induces modulation of the phytosterol pool in barley.**

Measurement of phytosterol content in mock-inoculated or *S. indica*-colonized barley roots at 3-, 7-, and 14-days post inoculation (dpi). The amounts of stigmasterol (stigmasta-5,22-dien-3β-ol) (A) and β-sitosterol (24β-ethylcholest-5-en-3β-ol) (B) were measured via GC-TOF-MS and the ratio of stigmasterol to β-sitosterol was calculated (C). Horizontal lines depict mean values. Letters indicate significant differences based on ANOVA and post hoc Tukey test (p ≤ 0.05). Relative response was calculated based on Internal standard response and normalized to sample fresh weight.

**
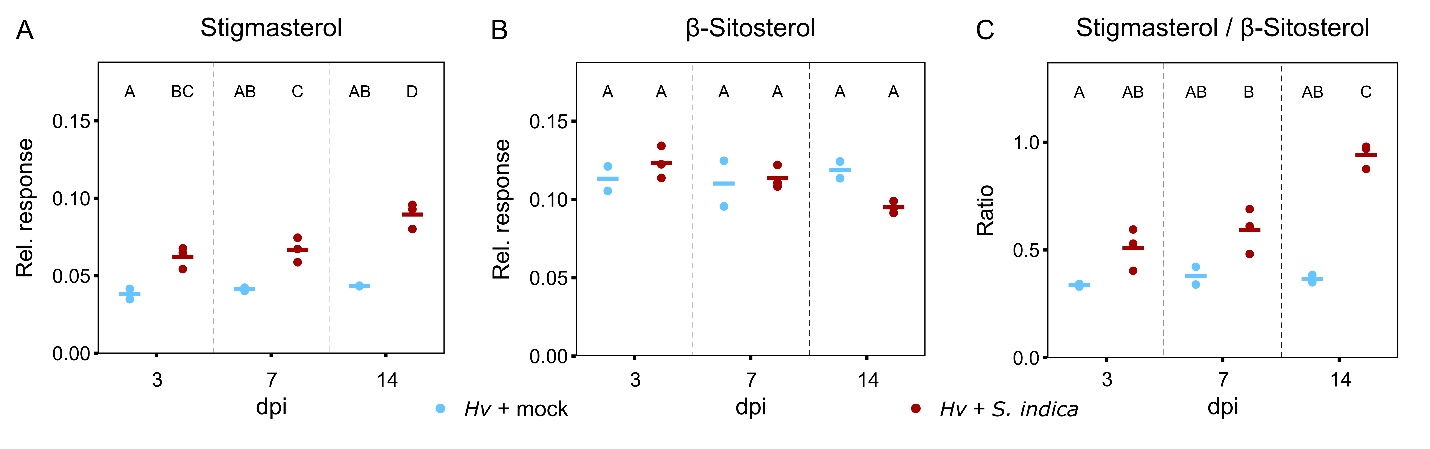
**

**Fig. S5: BAK1/SERK3 is not involved in *S. indica* lipid perception in *S. lycopersicum* leaves.**

A) ROS accumulation in *S. lycopersicum* cv Moneymaker WT and *S. lycopersicum* cv Moneymaker *serk3a serk3b* leaf discs, treated with the indicated elicitors or solvent control as negative control. Values represent means ± SEM from 12 wells, each containing one leaf disc. Two leaves were used per plant and three plants per genotype. The following concentrations/dilutions were used: ergosterol: 250 nM, flg22: 1 µM. All treatments contained 1:40 (v/v) final amount of methanol. B) Total ROS accumulation of the same experiment. Boxplots depict the interquartile range (IQR) ranging from the lower quartile Q1 (25th percentile) to the upper quartile Q3 (75th percentile). The horizontal line inside the box depicts the median. Data points outside 1.5 x IQR are depicted as outliers (thicker black dots). Asterisks indicate significant differences based on Student’s t-test (ns = not significant; p ≤ 0.05 *; p ≤ 0.01 **; p ≤ 0.001 ***, p ≤ 0.0001 ****). C) Pictures depicting the phenotype of the mutant plants compared to WT plants (curly leaves, reduced growth). D) Sequence alignment of the coding sequence of *SERK3A* and *SERK3B* downloaded from ensembl plant (Original sequence) and amplification of the respective genes from *S. lycorpersicum* cv Moneymaker WT gDNA (first 2 rows) and *S. lycopersicum* cv Moneymaker *serk3a serk3b* mutant gDNA (last 2 rows). Mutant plants contain a 7 AA deletion for *SERK3A* and a 6 AA deletion for *SERK3B*. RLU = Relative luminescence unit; ROS = Reactive oxygen species; SERK3 = Somatic embryogenesis receptor kinase 3; WT = Wildtype.

**
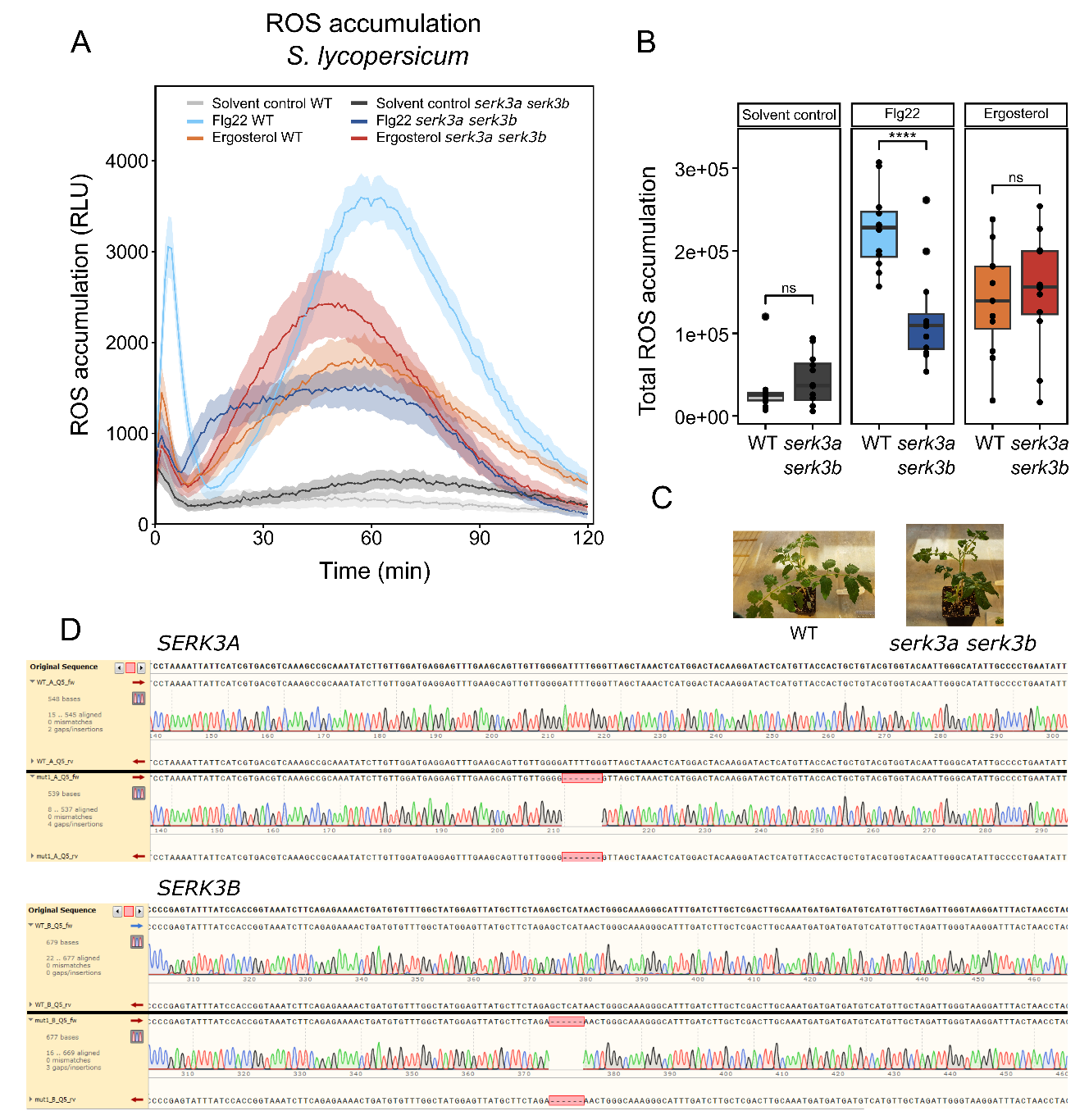
**

**Fig. S6: Phospholipid cotreatment does not enhance the chitohexaose-induced ROS burst in barley roots.**

**
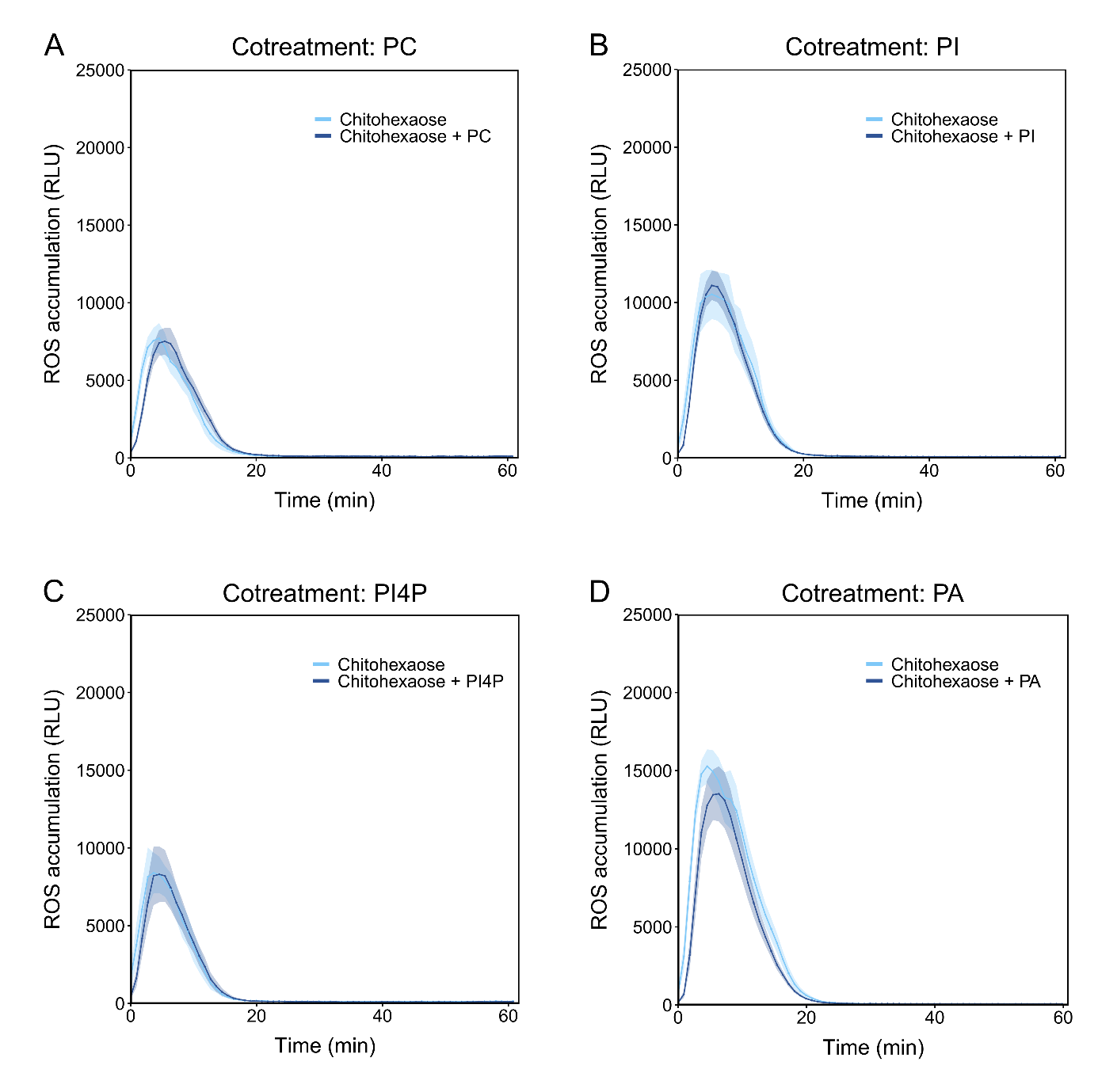
**A-D) ROS accumulation in roots of four days old barley plants, treated with the indicated elicitors or solvent control as negative control. Values represent means ± SEM from eight (without cotreatment) or sixteen (with cotreatment) wells, each containing three root pieces. The following concentrations and dilutions were used: chitohexaose: 250 nM and all phospholipids (PC, PI, PI4P, PA) were used at a final concentration of 250 nM. All treatments contained a final amount of 1:40 (v/v) methanol. PC = Phosphatidylcholine; PI = Phosphatidylinositol; PI4P = Phosphatidylinositol-4-phosphate; PA = Phosphatidic acid; RLU = Relative luminescence unit; ROS = Reactive oxygen species.

**Fig. S7: Cotreatment with PA liposomes enhances the ergosterol-induced ROS burst in barley roots.**

ROS accumulation in roots of four days old barley plants, treated with ergosterol or ergosterol + PA liposomes. Values represent means ± SEM from wells, each containing three root pieces. The following concentrations were used: Ergosterol: 250 nM, PA: 25µM. All treatments contained a final amount of 1:40 (v/v) methanol. PA liposomes were prepared in 25 mM HEPES buffer (pH 7.5, 50 mM KCL, 1mM MgCL_2_). Boxplots depict the interquartile range (IQR) ranging from the lower quartile Q1 (25th percentile) to the upper quartile Q3 (75th percentile). The horizontal line inside the box depicts the median. Data points outside 1.5 x IQR are depicted as outliers (thicker black dots). Asterisks indicate significant differences based on Student’s t-test (ns = not significant; p ≤ 0.05 *; p ≤ 0.01 **; p ≤ 0.001 ***, p ≤ 0.0001 ****). PA = Phosphatidic acid; RLU = Relative luminescence unit; ROS = Reactive oxygen species.

**
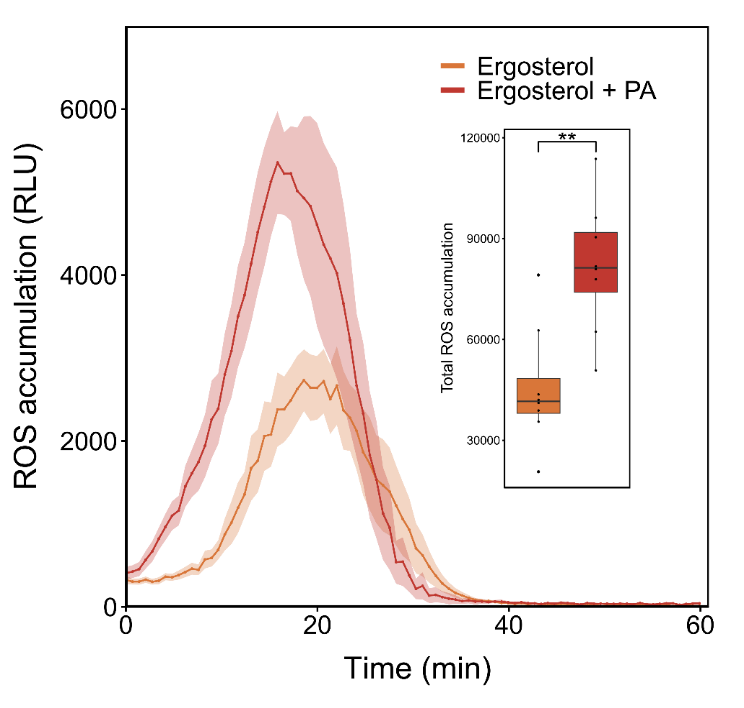
**

**Fig. S8: PA cotreatment does not enhance chitohexaose-induced ROS burst in barley roots.**

A-F) ROS accumulation in roots of four days old barley plants, treated with the indicated chitohexaose concentrations +/- 250 nM PA or solvent control as negative control. Values represent means ± SEM from eight (without cotreatment) or sixteen (with cotreatment) wells, each containing three root pieces. All treatments contained a final amount of 1:40 (v/v). PA = Phosphatidic acid; RLU = Relative luminescence unit; ROS = Reactive oxygen species.

**
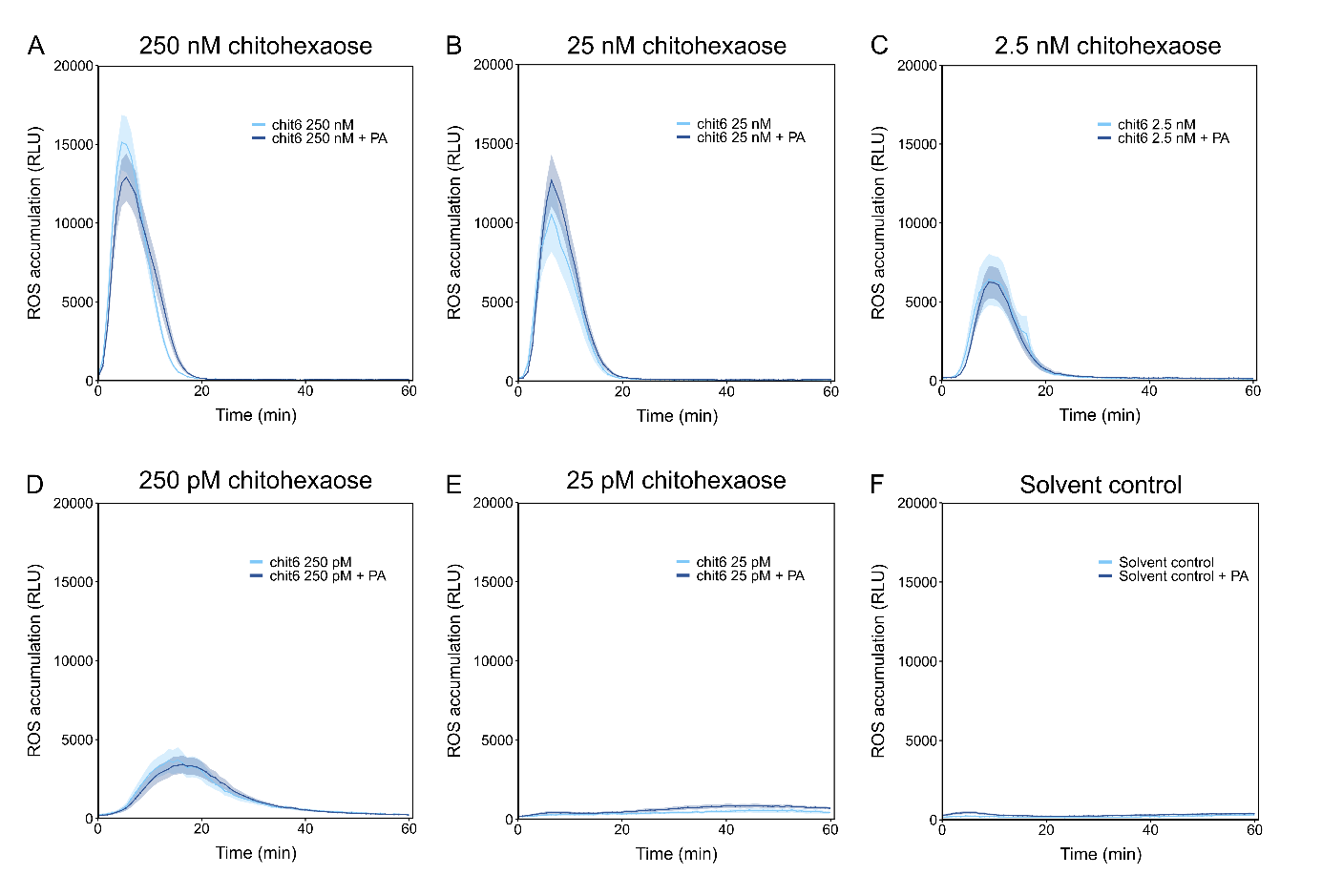
**

**Fig. S9: Diterpene exudation in response to ergosterol treatment and *B. sorokiniana* colonization.**

LC-MS/MS chromatogram of the second major diterpene found in root exudates of barley plants treated with solvent control or ergosterol or colonized by *B. sorokiniana* (positive control) for 6 days. The experiment was repeated with similar results. Ergosterol and solvent control treatments contained a final amount of 1:40 (v/v) methanol. *Hv* = *Hordeum vulgare* (barley).

**
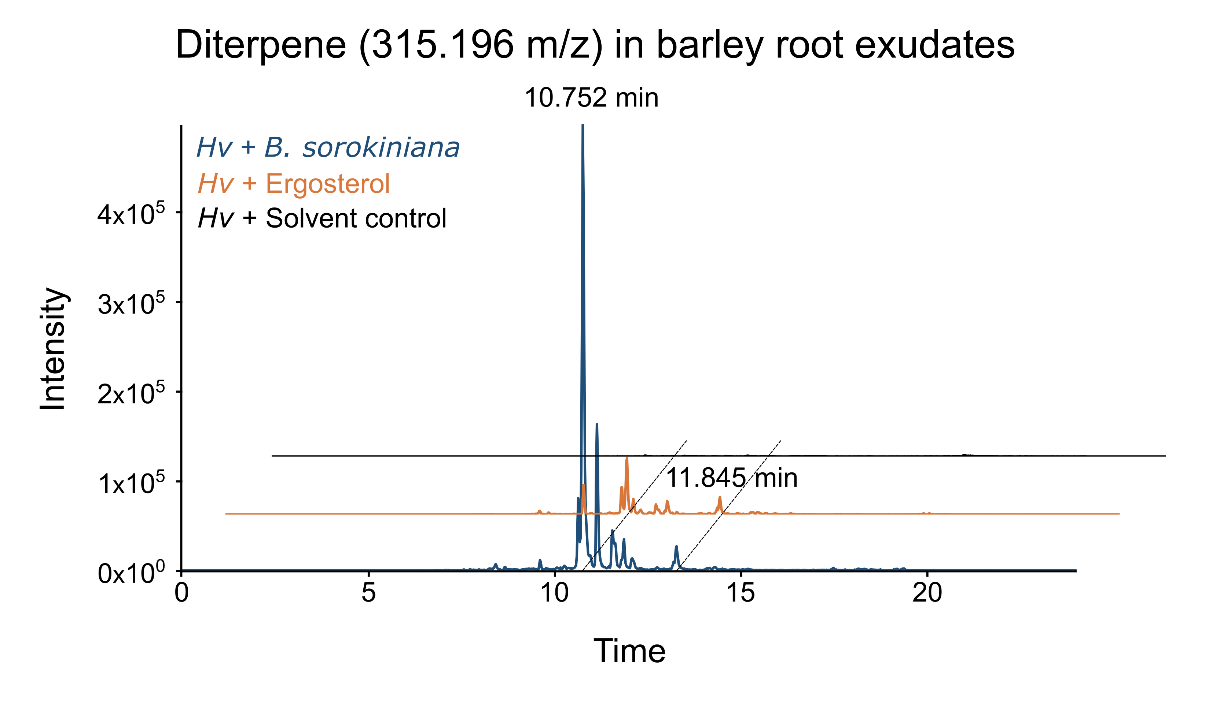
**

**Methods S1:** Detailed information on materials and methods.

**Plant material and growth conditions**

Seeds of *Hordeum vulgare* L. cv Golden Promise were surface sterilized with 6% sodium-hypochlorite, rotating for 1 h at 40 rpm and washed six times for 30 min with sterile milliQ-water. Seed coats were removed gently in sterile water using forceps and seeds were placed on wet filter paper (2 ml milliQ-water per 75 mm round filter paper) in a petri dish (eight seeds per filter paper). Plates were covered with aluminum foil and kept in the dark at 21 °C for three days before transfer to sterile WECK jars containing 100 ml solid 1/10 PNM (plant nutrition medium, pH 5.6) and 0.4 % (w/v) Gelrite for MAMP treatments and 1.2 % (w/v) Gelrite for fungal colonization in a growth chamber under long-day conditions (day/night cycle of 16/8 h, 22 °C/18 °C, light intensity of 108 μmol/m^2^*s). For MAMP treatment, seedlings were grown for an additional four days and for fungal colonization for 3-, 7- and 14- days.

For Ca^2+^-influx assays, barley lines expressing apoaequorin were generated. Therefore, *Aequorea victoria* aequorin was amplified from transgenic *A. thaliana* expressing cytosolic apoaequorin (Knight et al., 1991) with primers AEQ_Gib_Fw and AEQ_Gib_Rev (Supplementary Table S3) and cloned via Gibson assembly into HindIII-cut CPGE VEC00103 plasmid, which was then cut with SfiI+AhdI to allow separation of the fragments on gel and The resulting fragment with the *Sfi*I-ends (2918bp) containing the AEQ CDS under control of maize UBI promotor and NosT in between LB-RB was ligated into the SfiI-cut binary vector CPGE_VEC00006 and transformed into barley cv Golden Promise via embryo transformation (Hensel et al., 2009). Successful insertion of the transformation vector into the genome was tested by PCR (primers: Hv_AEQ_Fw, Hv_AEQ_Rev, Supplementary Table S3) on T0 plants. Positive offspring (T1) of 3:1 segregating lines were selected and further propagated to gain stable insertion lines (T2). Insertion of aequorin was verified by PCR (see above) and measuring discharge as described for the calcium assay and Plants with highest discharge levels were selected.

*Solanum lycopersicum* L. cv Moneymaker wildtype (WT) and *serk3a serk3b* mutant seeds were sown on soil and grown for ~2-3 weeks in the greenhouse under long-day conditions (day/night cycle of 17/7 h, 25-28 °C, ~35-40% humidity). For ROS assays, 3mm leaf discs of the two youngest, adult leaves of three weeks old tomato plants were used. *serk3a serk3b* mutant lines were generated using CRISPR/Cas9. A construct containing two sgRNAs, both of which target *Sl*SERK3A and *Sl*SERK3B was transformed (5' sgRNA AAGCAGTTGTTGGGGATTTT, 3' sgRNA ATGCTTCTAGAGCTCATAAC). Both sgRNAs target the penultimate exon within the cytoplasmic domain. Editing events were confirmed by Sanger sequencing.

Seeds of a *Nicotiana benthamiana* D. line expressing aequorin (Nb^AEQ^) (Wanke et al., 2020) were sown on soil and grown for ~3 weeks in the greenhouse under long-day conditions (day/night cycle of 16/8 h, 22–25°C, light intensity of ~140 μmol/m^2^*s, maximal humidity of 60%). For ROS and Ca^2+^-influx assays, 3mm leaf discs of the youngest, adult leaf of 3 weeks old plants were used.

Seeds of *Arabidopsis thaliana* L. Col-0 plants expressing cytosolic apoaequorin (At^AEQ^) were surface sterilized by incubation in 70 % EtOH for 10 min, followed by incubation in 100 % EtOH for 7 min. Subsequently, the EtOH was removed, and seeds dried under the clean bench. Dried seeds were placed on ½ MS (Murashige-Skoog-Medium, with vitamins, pH 5.7) plates containing 0.5 % sucrose and 0.4 % (w/v) Gelrite and covered with aluminum foil and stratified for 2-5 days at 4 °C. Plates were moved to the growth chamber with short day conditions (8 h light, 16 h dark, with 130 μmol/ m^2^*s of light and 22 °C/18 °C) and plants grown for 7-8 days. For ROS assays, one seedling was transferred into each well of a 96 well plate and the assay was performed as described below. For Ca^2+^-assays, seedlings were transferred to 24 well plates containing 1 ml ½ MS medium + sucrose and grown for another 4 days prior to use in the assay.

**Cultivation of *S. indica* and plant colonization**

Cultivation and chlamydospore isolation of *Serendipita indica* (DSM11827) was done as previously described (Sarkar et al., 2019; Wawra et al., 2016). For propagation, a stamp of 4 mm containing actively growing mycelium was removed from a 4-week-old plate with CM medium containing 1.5 % agar, transferred to a fresh plate and grown at 28 °C in darkness for 21 days prior to chlamydospore isolation for inoculation. Chlamydospores were collected in sterile milliQ water by gently scraping the surface of the mycelium on plate using a scalpel and filtered through Miracloth (Merck; 22-25 µm mesh size). Spores were pelleted by centrifugation at 2000 x g for 7 min, washed 3x in sterile milliQ water and diluted to a final concentration of 500.000 spores/ml for inoculations. Spores were counted using the Neubauer improved counting chamber.

To grow *S. indica* mycelium in axenic culture, 100 ml liquid CM medium was inoculated with 5 ml *S. indica* chlamydospores isolated from one 28 days old plate (approximately 1 million spores per ml) and grown at 28 °C with shaking at 120 rpm in a 500 ml Erlenmeyer flask for 5 days. Mycelium was then harvested in a Miracloth filter (Merck; 22-25 µm mesh size), surface-washed twice with 50 ml 0.9% NaCl and homogenized in a blender containing ~20 ml CM medium and transferred into fresh 150 ml CM medium and grown for 2 days. Mycelium was harvested and washed again and dried on tissue paper and snap frozen in liquid nitrogen.

For barley colonization, germinated seedlings were prepared as described above and inoculated with 3 ml of either sterile water as control or *S. indica* chlamydospores (500,000 spores/ml). Roots were harvested at 3-, 7- and 14 days post inoculation (dpi), washed thoroughly in ice-cold water to remove extraradical fungal hyphae, dried on tissue paper and frozen in liquid nitrogen. Four barley plants were used per jar and pooled per biological replicate.

**ROS accumulation assay**

ROS assays were performed as described previously (Chandrasekar et al., 2022). Preparation of *N. benthamiana*, tomato and *A. thaliana* material is described above. For barley, roots and shoots of seven days old seedlings were separated. The root tissue between 2 cm below the seed and 1 cm above the tip was cut into root pieces of 5 mm length and three root pieces transferred to each well of a 96-well microtiter plate (white, flat bottom) containing 200 μl of 2.5 mM MES buffer, pH 5.6. For barley shoot assays, 3 mm leaf discs were punched from one barley leaf per plant and one leaf-disc was transferred to each well of a similar 96-well microtiter plate. The plate was covered and incubated ON at 21°C for recovery. The next day, the buffer was replaced with 100 µl 2.5 mM MES buffer containing 20 µM LO-12 and 20 µg/ml HRP. After 25 min incubation in the dark, 100 µl two-fold concentrated elicitor solution or solvent control was added to each well and chemiluminescence was measured using a TECAN SPARK 10M microplate reader over all wells for 2h with an integration time of 450 msec.

**Calcium influx assay**

Preparation of *N. benthamiana* and *A. thaliana* material is described above. For barley, roots and shoots were prepared as described for ROS accumulation assays but plants expressing aequorin (Hv^AEQ^) were used. Prior to ON recovery, the buffer in the wells was replaced with 100 µl 2.5 mM MES buffer containing 10 µM coelenterazine and 10 mM CaCl_2_ per well and plates were incubated ON in the dark at 21°C. On the next day, chemiluminescence was measured using a TECAN SPARK 10M microplate plate reader. After the baseline measurement (5 min), 100 µl of two-fold concentrated elicitor solution was added manually. Photon emission was constantly measured for 30 minutes. Subsequently, 100 µl of discharge solution (3M CaCl_2_ in 50 % EtOH) was injected into each well, followed by constant measurement for 1 minute. All steps were performed with an integration time of 450 msec. In all assays, 2 columns (16 wells) were measured per run.

**MAPK phosphorylation**

Barley root segments and leaf discs were prepared as described above for ROS and Ca^2+^ influx assays. Twenty-four randomly selected pieces were transferred into each well of a 24-well plate containing 1 ml 2.5 mM MES buffer. Plates were incubated ON at 21°C for recovery. On the next day, 500 µl buffer of each well were removed and replaced with two-fold concentrated elicitor solution and gently mixed. At 5-, 10-, 20- or 30-min post treatment, roots were removed from the treatment solution, gently dried on tissue paper and snap frozen in liquid nitrogen and homogenized with glass beads in the TissueLyserII (Qiagen) for 4 times 30 sec, 30 Hz in ice-cold holders. For isolation of phosphorylated proteins, 150 µl phosphoprotein extraction buffer (50 mM Tris-HCl (pH 7.5), 2 mM DTT, 5 mM EDTA, 5 mM EGTA, 10 mM NaF, 50 mM β-glycerolphosphate, 10 % glycerol, 1 tablet each of Roche Complete Mini – EDTA free and PhosStop phosphatase inhibitor per 10 ml) was added, vortexed and centrifuged for 10 min at 4 °C at 13800 x g. The supernatant was transferred to a fresh tube and the centrifugation step was repeated two more times to ensure the extract is free of pellet debris. Protein concentration was determined using Bradford Assay following the manufacturer’s instructions and 4 µg protein of each sample were separated on SDS-PAGEs and subsequently transferred to nitrocellulose membranes. Membranes were blocked for 1h with 2.5 % TBS-T BSA and incubated ON with the primary antibody (anti-p44/p42, 1:1500) in 2.5 % TBS-T BSA at 4 °C. The next day, the membranes were washed with 1xTBS-T and the secondary antibody (anti-rabbit IgG, 1:50.000) was added for 1h. After washing with 1xTBS-T and 1x TBS, blots were developed using 1ml SuperSignal™West Femto (Thermo Scientific) solution per membrane. Western Blots were imaged using the Fujifilm LAS 4000 mini camera.

**Elicitor preparations**

The following chemicals were used as elicitors, pre- or cotreatments. chitohexaose (Megazyme, O-CHI6), flg22 peptide (GenScript, RP19986), ergosterol pharmaceutical standard (Supelco, PHR1512), L-α-phosphatidylinositol (soy PI, Avanti, 840044), L-α-phosphatidic acid (soy PA, Avanti, 840074), L-α-phosphatidylcholine (soy PC, Avanti, 441601), L-α-phosphatidylinositol 4-monophosphate (Sigma-Aldrich, P9638), L-α-phosphatidylinositol 4,5-diphosphate (Sigma-Aldrich, P9763). Stock solutions of lipids were prepared in methanol and used to prepare elicitor solutions of appropriate concentrations in aqueous MES buffer as described below. Self-produced lipid extracts and lipid fractions were evaporated with N_2_ gas and resuspended in MeOH as solvent. As control, the respective solvent was processed in the same way. All elicitor and control solutions were prepared as two-fold concentrated solutions in 2.5 mM MES buffer, pH 5.6 containing 1:20 (v/v %) MeOH. For liposomes, PA was evaporated under N_2_ gas and the lipid film hydrated with buffer (25 mM HEPES, pH=7.5, 50 mM KCL, 1 mM MgCL_2_) at 4°C, rapidly vortexed for 30 seconds and sonicated on ice 5 times for 10 sec with each 10 sec pause prior to use in cotreatment ROS assay with ergosterol.

**RNA-seq and qRT-PCR**

For RNA-seq and qRT-PCR, barley roots were prepared as described for the MAPK phosphorylation assay with roots from two wells being combined per replicate and four replicates per treatment and timepoint. RNA extraction, cDNA synthesis and qRT-PCR were performed as described previously (Sarkar et al., 2019). Briefly, RNA was extracted using Trizol (Life Technologies, 15596018) and remaining DNA was removed by DNAseI (Thermo Scientific, EN0521) digestion at 37 °C for 30 min. One µg RNA was used to synthesize cDNA using the first strand cDNA kit (Thermo Scientific, K1612) according to the manufacturer’s instructions. GoTaq qPCR Master Mix (Promega A60001) was used for qRT-PCR. Primers used for qRT-PCR are described in Supplementary Table S3.

***RNA sequencing and data processing***

For RNA sequencing, 25 µl RNA with a concentration of 100 ng/µl was used. For Illumina-compatible RNAseq libraries at first an enrichment for poly-A RNAs was performed (NEBNext® Poly(A) mRNA Magnetic Isolation Module; New England Biolabs), followed by library generation with NEBNext Ultra™II Directional RNA Library Prep Kit for Illumina (New England Biolabs). Next, sequencing-by-synthesis was done on a NextSeq 2000 device in 2 x 150 bp paired-end read mode Library construction and sequencing was performed at the Genome Centre of the Max Planck Institute for Plant Breeding, Cologne. Trimmomatic (v. 0.39) (Bolger et al., 2014) was used for quality trimming and adapter clipping and quality of the reads was assessed using fastQC. Reads were then mapped to the barley Morexv3 pseudomolecules assembly cDNA (downloaded from Ensembl plants on 18.08.2023) and quantified using kallisto (v. 0.46.1) (Bray et al., 2016), which resulted in estimated counts and transcripts per million (TPM) values. The log2 fold difference of the gene expression between conditions was calculated using R and differential expression of genes was calculated using the R package “sleuth”.

**Apoplastic fluid isolation of barley roots**

To isolate apoplastic fluid from barley roots, barley seedlings were grown and inoculated with *S. indica* on 1/10 PNM medium as described above. Per replicate, 110 barley seedlings were used, which yielded approximately ~1 ml apoplastic fluid per replicate. For extraction of apoplastic fluid, the roots were gently removed from the jars and washed thoroughly in ice-cold water to remove external fungal hyphae. The upper and lower 1 cm of the root were cut off and the remaining root was cut into 2 cm pieces. Root pieces of one replicate were transferred into two 50 ml falcon tubes containing approx. 35 ml of ice-cold water and covered with ice. Next, falcon tubes were submitted to five cycles of vacuum infiltration (15 min 250 mbar, 1.5 min ATM). Subsequently, roots were dried on tissue paper and transferred into a 20 ml syringe inside a 50 ml falcon tube and centrifuged for 15 min at 4 °C, 711 x g, lowest de- and acceleration to collect apoplastic fluid (approx. 1-2 ml per replicate) in the bottom of the falcon tube. Apoplastic fluid was stored on ice at 4 °C until further use. Roots were flash-frozen in liquid nitrogen and stored at -80 °C until further use.

**Lipid extraction and fractionation by solid phase extraction**

Lipid extraction was done according to the method described by Bligh and Dyer (Bligh & Dyer, 1959). To extract lipids from *S. indica* mycelium or colonized or mock-inoculated barley roots, frozen material was ground in liquid nitrogen into a very fine powder using mortar and pestle. Per sample, ~200 mg of homogenized plant or fungal tissue was transferred into a pre-cooled 2 ml Eppendorf tube and 1 ml (2 vol) chloroform:methanol:formic acid (1:1:0.1 v/v) was added to the Eppendorf tube. To isolate lipids from apoplastic fluid, ~1 ml apoplastic fluid was added to a 5 ml Eppendorf tube and 2 ml (2 vol) chloroform:methanol:formic acid (1:1:0.1 v/v) was added. The mixture was vortexed and incubated for 10 min at RT, shaking. Next, 0.5 ml (1 vol) of 300 mM ammonium acetate was added to create a phase separation and the samples were vortexed again, followed by centrifugation for 5 min at 3500 x g. The lower organic phase was transferred to a fresh glass tube using a glass Pasteur pipette that was previously rinsed with chloroform. 0.8 ml chloroform was added again to each tube, vortexed and centrifuged for 5 min at 3500 x g. The lower organic phase was transferred again and combined with the first extract. This step was repeated two more times. The crude lipid extract was evaporated under N_2_ gas and resuspended in 4 ml MeOH for use in preparation of elicitor treatment with crude lipids.

For fractionation of crude lipids, the lipid extract was separated by two consecutive solid phase extractions (SPE). First, the crude lipid extract was dried under N_2_ gas and resuspended in 1 ml chloroform and added to a Strata®SI-1 (55 µm, 70 Å, 1 ml) silica column, previously equilibrated with chloroform. Two times 1 ml chloroform was added again to the silica column and the eluate, containing neutral lipids, collected in the same tube. Then, three times 1 ml MeOH was added to the column and the fraction containing polar (phospho)lipids collected. The chloroform fraction was evaporated under N_2_ gas and used for further fractionation of the neutral lipids using a hexane diethylether gradient on a second silica column as described previously (vom Dorp *et al.*, 2013). In brief, the dried lipids were resuspended in 100 % hexane and added to the column, previously equilibrated with hexane, and a total of six fractions were collected by adding each three column volumes (1 ml) of stepwise decreasing hexane:diethylether ratios (v:v): 100:0 (hydrocarbons and squalene), 98:2 (sterol esters), 95:5 (triacylglycerol, alkyl and alkenyl acylglycerols and tocopherols; free fatty acids and fatty alcohols), 85:15 (free sterols and diacylglycerol) and finally 0:100 (diacylglycerol and monoacylglycerol). All fractions that were used in immunity assays were dried under N_2_ and resuspended in 4 ml MeOH prior to usage

**Phosphoproteomics**

***Preparation of phospho-enriched samples***

Barley roots were treated as described for MAPK phosphorylation assay. Two wells were combined for each replicate per treatment and time point. Four replicates were used each. Root material was harvested 10 min post treatment. Root material was ground using mortar and pestle with liquid nitrogen into a very fine powder and 1 ml extraction buffer (8M urea, 20 µl/ml Phosphatase Inhibitor Cocktail 2 (Sigma, P5726-5ML), 20 µl/ml Phosphatase Inhibitor Cocktail 3 (Sigma, P0044-5ML), 5 mM DTT) was added and samples were incubated for 30 min with shaking, after which cell debris was removed by centrifugation. Samples were alkylated with CAA (550 mM stock, 14 mM final), the reaction was quenched with DTT (5 mM final). An equivalent of 500 µg total protein per sample was diluted to 1 M urea with 100 mM Tris-HCl pH 8.5, 1 mM CaCl_2_ and samples were digested with 5 µg LysC (stock: 1 µg/µl Lys-C (WAKO) in 50 mM NH_4_HCO_3_) for 4h at RT. Next, 5 µg trypsine (stock: 1 µg/µl in 1 mM HCl,) was added and samples were diluted with 100 mM Tris-HCl pH 8.5, 1 mM CaCl_2_, the samples were mixed and incubated ON at 37 °C. After incubation, samples were acidified with TFA to 0.5 % final concentration and samples were desalted using C18 SepPaks (1cc cartridge, 100 mg (WAT023590)). In brief, SepPaks were conditioned using methanol (1 ml), buffer B (80% acetonitrile, 0.1% TFA) (1 ml) and buffer A (0.1% TFA) (2 ml). Samples were loaded by gravity flow, washed with buffer A (1 x 1 ml, 1x 2 ml) and eluted with buffer B (2 x 400 µl). 44 µl of eluates were used for peptide measurement and total proteome and library analysis. For phosphopeptide enrichment by metal-oxide chromatography (MOC) (adapted from:(Nakagami, 2014)) the remaining samples were evaporated to a sample volume of 50 µl and diluted with sample buffer (2 ml AcN, 820 µl lactic acid (LA), 2.5 µl TFA / 80 % ACN, 0.1 % TFA, 300 mg/ml LA, final concentrations) (282 µl). MOC tips were prepared by loading a slurry of 3 mg/sample TiO_2_ beads (Titansphere TiO_2_ beads 10 µm (GL Science Inc, Japan, Cat. No. 5020-75010)) in 100 µl MeOH onto a C8 micro column and centrifugation for 5 min at 1500g. Tips were washed with centrifugation at 1500g for 5 min using 80 µl of solution B (80 % acetonitrile, 0.1 % TFA) and 80 µl of solution C (300 mg/ml LA in solution B). To simplify the processing, samples tips were fitted onto a 96/500 µl deep well plate (Protein LoBind, (Eppendorf Cat. No. 0030504100). After washing MOC tips were transferred to a fresh plate, samples were loaded onto the equilibrated tips and centrifuged for 10 min at 1000g. The flow through was reloaded onto the tips and centrifugation was repeated. Tips were washed with centrifugation at 1500g for 5 min using 80 µl of solution C and 3x 80 µl of solution B. For the elution of the enriched phosphopeptides the tips were transferred to a fresh 96/500 µl deep well plate containing 100 µl/well of acidification buffer (20 % phosphoric acid). Peptides were eluted first with 50 µl elution buffer 1 (5% NH_4_OH) and centrifugation for 5 min at 800g, then with 50 µl of elution buffer 2 (10% piperidine) and centrifugation for 5 min at 800g. Next, the samples were desalted using StageTips with C18 Empore disk membranes (3 M) (Rappsilber et al., 2003), dried in a vacuum evaporator, and dissolved in 10 µl 2% ACN, 0.1% TFA (A* buffer) for MS analysis.

***LC-MS/MS data acquisition***

Samples were analyzed using an Ultimate 3000 RSLC nano (Thermo Fisher) coupled to an Orbitrap Exploris 480 mass spectrometer equipped with a FAIMS Pro interface for Field asymmetric ion mobility separation (Thermo Fisher). Peptides were pre-concentrated on an Acclaim PepMap 100 pre-column (75 µM x 2 cm, C18, 3 µM, 100 Å, Thermo Fisher) using the loading pump and buffer A** (water, 0.1 % TFA) with a flow of 7 µl/min for 5 min. Peptides were separated on 16 cm frit-less silica emitters (New Objective, 75 µm inner diameter), packed in-house with reversed-phase ReproSil-Pur C18 AQ 1.9 µm resin (Dr. Maisch). Peptides were loaded on the column and eluted for 130 min using a segmented linear gradient of 5 % to 95 % solvent B (0 min : 5 %B; 0-5 min -> 5 %B; 5-65 min -> 20 %B; 65-90 min ->35 %B; 90-100 min -> 55 %; 100-105 min ->95 %, 105-115 min ->95 %, 115-115.1 min -> 5 %, 115.1-130 min ->5 %) (solvent A 0 % ACN, 0.1 % FA; solvent B 80 % ACN, 0.1 %FA) at a flow rate of 300 nl/min. Mass spectra were acquired in data-dependent acquisition mode with a TOP_S method using a cycle time of 2 seconds. For field asymmetric ion mobility separation (FAIMS) two compensation voltages (-45 and -65) were applied, the cycle time for the CV-45 experiment was set to 1.2 seconds and for the CV-65 experiment to 0.8 sec. MS spectra were acquired in the Orbitrap analyzer with a mass range of 320–1200 m/z at a resolution of 60,000 FWHM and a normalized AGC target of 300 %. Precursors were filtered using the MIPS option (MIPS mode = peptide), the intensity threshold was set to 5000, Precursors were selected with an isolation window of 1.6 m/z. HCD fragmentation was performed at a normalized collision energy of 30 %. MS/MS spectra were acquired with a target value of 75 % ions at a resolution of 15,000 FWHM, at an injection time of 120 ms and a fixed first mass of m/z 120. Peptides with a charge of +1, greater than 6, or with unassigned charge state were excluded from fragmentation for MS^2^.

***Data analysis of phosphoproteomics***

Raw data were processed using MaxQuant software (version 1.6.3.4, <http://www.maxquant.org/>) (Cox & Mann, 2008) with label-free quantification (LFQ) and iBAQ enabled (Tyanova et al., 2016).

MS/MS spectra were searched by the Andromeda search engine against a combined database containing the sequences from *H. vulgare* (Morex V3 database), and sequences of 248 common contaminant proteins and decoy sequences. Trypsin specificity was required and a maximum of two missed cleavages allowed. Minimal peptide length was set to seven amino acids. Carbamidomethylation of cysteine residues was set as fixed, phosphorylation of serine, threonine and tyrosine, oxidation of methionine and protein N-terminal acetylation as variable modifications. The match between runs option was enabled. Peptide-spectrum-matches and proteins were retained if they were below a false discovery rate of 1 % in both cases.

Statistical analysis was carried out on phospho peptide level using the intensities obtained from the “modificationSpecificPeptides” output using Perseus (version 1.6.14.0, <http://www.maxquant.org/>). Quantified sites were filtered for reverse hits and contaminant hits and results were filtered to retain only phospho-modified peptides. Intensities were log2 transformed and samples were grouped by condition. Next, the data was separated for a mixed imputation processing: hits were filtered for 3 valid values in one of the conditions and intensity values were normalized by subtraction of the median from each column. Then, the data was separated into two sets: one set containing mostly missing at random (MAR) hits and the other set containing mostly missing not at random (MNAR) hits by filtering the data for 1 valid hit in each group and splitting the resulting matrices (Lazar et al., 2016). The resulting matrix with at least 1 valid hit in each group is the MAR dataset, the matrix with the hits filtered out is the MNAR dataset. The missing values of each dataset were then imputed using different options of the “imputeLCMD” R package (Cosmin Lazar (2015). imputeLCMD: A collection of methods for left-censored missing data imputation. R package version 2.0. <http://CRAN.R-project.org/package=imputeLCMD>) integrated into Perseus: the missing values from the MAR dataset were imputed using a nearest neighbor approach (KNN, n=4), the missing values from the MNAR dataset were imputed using the MinProb option (q=0.01. tune.sigma=1). After merging of the imputed datasets two-sample Student’s *t*-tests were performed using a permutation-based FDR of 5%. Alternatively, volcano plots were generated using an FDR=0.05 and an S0=1. The Perseus output was exported and further processed using Excel. For downstream analyses the imputed as well as the MNAR datasets were used.

**Measurements of sterols via GC-TOF-MS**

For sterol measurement via GC-TOF-MS, 5 nmol stigmastanol was added as internal standard in a chloroform:MeOH (2:1) mix prior to lipid extraction. Lipid extraction and SPE was performed as described above. Dried free sterol fractions were directly derivatized with 100 µl MSTFA for 30 min at 80 °C. Subsequently the samples were transferred to glass vials and measured in a 1:2 dilution with split ratio of 1:10. To measure crude lipid extracts of apoplastic fluid, dried lipid extracts were resuspended in 300 µl hexane and split in 2x 150 µl samples. One 150 µl sample each was evaporated under N_2_ gas. Dried samples were derivatized with MSTFA for 30 min at 80°C automatically prior to measurement using a Multipurpose Autosampler (Gerstel). 1 µl of sample was injected with an automatic liner exchange system in conjunction with a cold injection system in splitless mode (ramping from 50 °C to 250 °C at 12 °C s-1) into the GC with a helium flow of 1 ml/min. Chromatography was performed using a 7890B GC system (Agilent Technologies) with a HP-5MS column with 5 % phenyl methyl siloxane film (Agilent 19091S-433, 30 m length, 0.25 mm internal diameter, 0.25 µM film). The oven temperature was held constant at 70 °C for 1 min and then ramped at 42 °C min-1 to 280 °C and in a second step with 4 °C min-1 to 320 °C which was held for 3 min resulting in a total run time of 19 minutes. Samples were ionized with an electron impact source at 70 eV and 200 °C source temperature and recorded in a mass range of m/z 60 to m/z 800 at 20 scans per second with a 7200 GC-QTOF (Agilent Technologies).
Compounds were identified via MassHunter Qualitative (v b08.00, Agilent Technologies) by comparison of spectra to the NIST14 Mass Spectral Library (https://www.nist.gov/srd/nist-standard-reference-database-1a-v14). Ergosterol was verified using a pharmaceutical standard (Supelco, PHR1512) and stigmastanol was used as an internal standard. Peaks were integrated using MassHunter Quantitative (v b08.00, Agilent Technologies). For relative quantification, all metabolite peak areas were normalized to the corresponding peak area of the internal standard stigmastanol and the sample fresh weight (mycelium, roots) or volume (AF).

**Diterpene measurements via LC-MS**

To measure diterpenes in root exudates of barley plants, seedlings were transferred into WECK jars containing 100 ml 1/10 PNM solution and 3 ml sterile water was added to the roots. Seedlings were incubated ON in growth chamber and 5 ml treatment solution (solvent control, 250 nM Ergosterol or *Bipolaris sorokiniana* spores (5000 spores/ml, 5ml) and) were added on the next day and grown for 6 days. To harvest root exudates, plants were removed from the medium and washed gently in 25 ml water to wash off residual diterpenes attached on the outside of the roots. The medium was collected with the wash water from the roots and flash-frozen in liquid nitrogen. Diterpenes were extracted as described previously (Liu et al., 2024). Briefly, root exudates were extracted two times with a mixture of 70 % ethylacetate and 30 % hexane (v/v) by vigorous shaking. After phase separation the organic phase was collected. 150 mg of plant matter was extracted in a modified Bligh and Dyer extraction in two rounds with 900 µl of a mixture of 67 % dichloromethane and 33 % ethanol and 150 µl of hydrochloric acid of pH 1.4. The organic phase containing diterpenoids was collected. All organic extracts were dried in nitrogen stream and stored at -80 °C until analysis.

Separation of medium polar metabolites was performed on a Nucleoshell RP18 (2.1 x 150 mm, particle size 2.1 µm, Macherey & Nagel, GmbH, Düren, Germany) using a Waters ACQUITY UPLC System, equipped with an ACQUITY Binary Solvent Manager and ACQUITY Sample Manager (20 µl sample loop, partial loop injection mode, 5 µl injection volume, Waters GmbH Eschborn, Germany). Eluents A and B were aqueous 0.3 mmol/*l* NH_4_HCOO (adjusted to pH 3.5 with formic acid) and acetonitrile, respectively. Elution was performed isocratically for 2 min at 5 % eluent B, from 2 to 19 min with linear gradient to 95 % B, from 19-21 min isocratically at 95 % B, and from 21.01 min to 24 min at 5 % B. The flow rate was set to 400 µl/min and the column temperature was maintained at 40 °C.

Mass spectrometric analysis of small molecules was performed by MS1 full scan from 65-1500 Dalton and 100 ms accumulation time (ZenoToF 7600, AB Sciex GmbH, Darmstadt, Germany) operating in negative ion mode and controlled by Sciex OS software (Sciex). The declustering potential was set to -80 V with a spread of 50 V. MS/MS-CID fragmentation was triggered by data dependent acquisition in 20 ms pockets and up to 40 candidate spectra were recorded between 65-1500 Dalton for ions were the threshold exceeded 150 cps. As for MS1 the declustering potential was set to -80 V and a spread of 50 V, while the collision energy was set to -35 V and a spread of 25 V. The source operation parameters were as the following: ion spray voltage, -4500 V; nebulizing gas, 60 psi; source temperature, 600 °C; drying gas, 70 psi; curtain gas, 35 psi CAD gas 7 psi. Instrument tuning and internal mass calibration were performed every 5 samples with the calibrant delivery system applying X500 ESI negative tuning solution (AB Sciex GmbH, Darmstadt, Germany).MS1 data for selected mz;r.t. couples were integrated using MultiQuant TF (AB Sciex GmbH, Darmstadt, Germany) within a tolerance window of 10 ppm and a smooth factor of 1.0.

**References:**

Bligh, E. G., & Dyer, W. J. (1959). A Rapid Method of Total Lipid Extraction and Purification. *Canadian Journal of Biochemistry and Physiology*, *37*(8).

Bolger, A. M., Lohse, M., & Usadel, B. (2014). Trimmomatic: a flexible trimmer for Illumina sequence data. *Bioinformatics*, *30*(15), 2114–2120. https://doi.org/10.1093/BIOINFORMATICS/BTU170

Bray, N. L., Pimentel, H., Melsted, P., & Pachter, L. (2016). Near-optimal probabilistic RNA-seq quantification. *Nature Biotechnology 2016 34:5*, *34*(5), 525–527. https://doi.org/10.1038/nbt.3519

Chandrasekar, B., Wanke, A., Wawra, S., Saake, P., Charura, N., Neidert, M., Malisic, M., Thiele, M., Pauly, M., & Zuccaro, A. (2022). Fungi hijack a plant apoplastic endoglucanase to release a ROS scavenging β-glucan decasaccharide to subvert immune responses. *The Plant Cell*, *34*, 2765–2784. https://doi.org/10.1101/2021.05.10.443455

Cox, J., & Mann, M. (2008). MaxQuant enables high peptide identification rates, individualized p.p.b.-range mass accuracies and proteome-wide protein quantification. *Nature Biotechnology 2008 26:12*, *26*(12), 1367–1372. https://doi.org/10.1038/nbt.1511

Hensel, G., Kastner, C., Oleszczuk, S., Riechen, J., & Kumlehn, J. (2009). Agrobacterium-mediated gene transfer to cereal crop plants: current protocols for barley, wheat, triticale, and maize. *International Journal of Plant Genomics*, *2009*. https://doi.org/10.1155/2009/835608

Knight, M. R., Campbell, A. K., Smith, S. M., & Trewavas, A. J. (1991). Transgenic plant aequorin reports the effects of touch and cold-shock and elicitors on cytoplasmic calcium. *Nature 1991 352:6335*, *352*(6335), 524–526. https://doi.org/10.1038/352524a0

Lazar, C., Gatto, L., Ferro, M., Bruley, C., & Burger, T. (2016). Accounting for the Multiple Natures of Missing Values in Label-Free Quantitative Proteomics Data Sets to Compare Imputation Strategies. *Journal of Proteome Research*, *15*(4), 1116–1125. https://doi.org/10.1021/ACS.JPROTEOME.5B00981

Liu, Y., Esposto, D., Mahdi, L. K., Porzel, A., Stark, P., Hussain, H., Scherr-Henning, A., Isfort, S., Bathe, Ulschan., Acosta, I. F., Zuccaro, A., Balcke, G. U., & Tissier, A. (2024). Hordedane diterpenoid phytoalexins restrict Fusarium graminearum infection but enhance Bipolaris sorokiniana colonization of barley roots. *Molecular Plant*, *17*(8), 1307–1327. https://doi.org/10.1016/J.MOLP.2024.07.006

Nakagami, H. (2014). StageTip-based HAMMOC, an efficient and inexpensive phosphopeptide enrichment method for plant shotgun phosphoproteomics. *Methods in Molecular Biology (Clifton, N.J.)*, *1072*, 595–607. https://doi.org/10.1007/978-1-62703-631-3_40

Rappsilber, J., Ishihama, Y., & Mann, M. (2003). Stop And Go Extraction tips for matrix-assisted laser desorption/ionization, nanoelectrospray, and LC/MS sample pretreatment in proteomics. *Analytical Chemistry*, *75*(3), 663–670. https://doi.org/10.1021/AC026117I/ASSET/IMAGES/LARGE/AC026117IF00005.JPEG

Sarkar, D., Rovenich, H., Jeena, G., Nizam, S., Tissier, A., Balcke, G. U., Mahdi, L. K., Bonkowski, M., Langen, G., & Zuccaro, A. (2019). The inconspicuous gatekeeper: endophytic Serendipita vermifera acts as extended plant protection barrier in the rhizo-sphere. *New Phytologist*, *224*, 886–901. https://doi.org/10.1111/nph.15904

Tyanova, S., Temu, T., & Cox, J. (2016). The MaxQuant computational platform for mass spectrometry-based shotgun proteomics. *Nature Protocols 2016 11:12*, *11*(12), 2301–2319. https://doi.org/10.1038/nprot.2016.136

Wanke, A., Rovenich, H., Schwanke, F., Velte, S., Becker, S., Hehemann, J. H., Wawra, S., & Zuccaro, A. (2020). Plant species-specific recognition of long and short β-1,3-linked glucans is mediated by different receptor systems. *Plant Journal*, *102*(6), 1142–1156. https://doi.org/10.1111/tpj.14688

Wawra, S., Fesel, P., Widmer, H., Timm, M., Seibel, J., Leson, L., Kesseler, L., Nostadt, R., Hilbert, M., Langen, G., & Zuccaro, A. (2016). The fungal-specific β-glucan-binding lectin FGB1 alters cell-wall composition and suppresses glucan-triggered immunity in plants. *Nature Communications 2016 7:1*, *7*(1), 1–11. https://doi.org/10.1038/ncomms13188
